# Maternal Western diet exposure increases periportal fibrosis beginning in utero in nonhuman primate offspring

**DOI:** 10.1172/jci.insight.154093

**Published:** 2021-12-22

**Authors:** Michael J. Nash, Evgenia Dobrinskikh, Sean A. Newsom, Ilhem Messaoudi, Rachel C. Janssen, Kjersti M. Aagaard, Carrie E. McCurdy, Maureen Gannon, Paul Kievit, Jacob E. Friedman, Stephanie R. Wesolowski

**Affiliations:** 1 Department of Pediatrics, Section of Neonatology, University of Colorado Anschutz Medical Campus, Aurora, Colorado, USA.; 2 Department of Molecular Biology and Biochemistry, School of Biological Sciences, University of California, Irvine, Irvine, California, USA.; 3 Harold Hamm Diabetes Center, University of Oklahoma Health Sciences Center, Oklahoma City, Oklahoma, USA.; 4 Department of Obstetrics and Gynecology, Division of Maternal-Fetal Medicine, and Departments of Molecular and Human Genetics and Molecular and Cell Biology, Baylor College of Medicine, Houston, Texas, USA.; 5 Department of Human Physiology, University of Oregon, Eugene, Oregon, USA.; 6 Division of Diabetes, Endocrinology, and Metabolism, Department of Medicine, Vanderbilt University Medical Center, Nashville, Tennessee, USA.; 7 Division of Cardiometabolic Health, Oregon National Primate Research Center, Oregon Health & Science University, Beaverton, Oregon, USA.

**Keywords:** Gastroenterology, Obesity

## Abstract

Maternal obesity affects nearly one-third of pregnancies and is a major risk factor for nonalcoholic fatty liver disease (NAFLD) in adolescent offspring, yet the mechanisms behind NAFLD remain poorly understood. Here, we demonstrate that nonhuman primate fetuses exposed to maternal Western-style diet (WSD) displayed increased fibrillar collagen deposition in the liver periportal region, with increased *ACTA2* and *TIMP1* staining, indicating localized hepatic stellate cell (HSC) and myofibroblast activation. This collagen deposition pattern persisted in 1-year-old offspring, despite weaning to a control diet (CD). Maternal WSD exposure increased the frequency of DCs and reduced memory CD4^+^ T cells in fetal liver without affecting systemic or hepatic inflammatory cytokines. Switching obese dams from WSD to CD before conception or supplementation of the WSD with resveratrol decreased fetal hepatic collagen deposition and reduced markers of portal triad fibrosis, oxidative stress, and fetal hypoxemia. These results demonstrate that HSCs and myofibroblasts are sensitive to maternal WSD-associated oxidative stress in the fetal liver, which is accompanied by increased periportal collagen deposition, indicative of early fibrogenesis beginning in utero. Alleviating maternal WSD-driven oxidative stress in the fetal liver holds promise for halting steatosis and fibrosis and preventing developmental programming of NAFLD.

## Graphical abstract

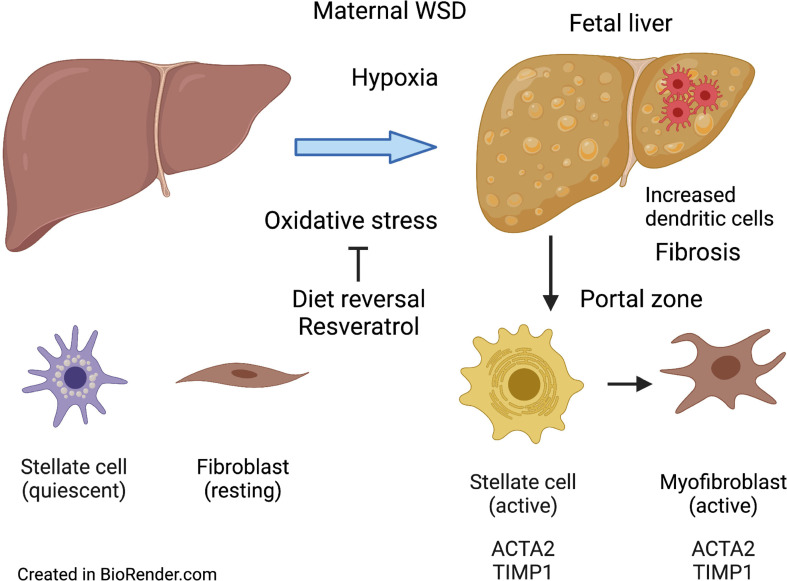

## Introduction

Obesity rates continue to increase and have reached epidemic proportions around the globe (1). Currently, nearly 50% of women who are pregnant in the US are overweight or obese (2–4). Accompanying maternal obesity is an increase in noncommunicable metabolic disorders in children, leading to a body of literature that suggests that metabolic disorders, including obesity, type 2 diabetes mellitus, cardiovascular disease, and nonalcoholic fatty liver disease (NAFLD), are often of intrauterine origin (4–6). Among these, NAFLD is the most common liver disease worldwide; it affects nearly 40% of obese youth and up to 10% of the general pediatric population (7). NAFLD is characterized by steatosis (excess liver fat) that over time may progress to nonalcoholic steatohepatitis (NASH), with accompanying inflammation and fibrosis, leading to cirrhosis and increased risk for hepatocellular carcinoma (8). NAFLD can progress rapidly in children, leading to end-stage liver disease and liver transplantation in early adulthood or sooner (4, 9) for reasons that remain poorly understood.

Despite advances in adults, major gaps remain in defining the pathways and mechanisms unique to childhood-onset NAFLD pathology (4, 6). Cohort studies have described early life risk factors for NAFLD, which include in utero exposure to maternal obesity and accelerated growth in childhood (10). In the NASH Clinical Research Network multicenter, cross-sectional study of children with NAFLD, children with high birth weight had significantly greater odds of severe steatosis and NASH even after controlling for childhood BMI, while those with low birth weight were more likely to have advanced fibrosis, suggesting that early exposures play an important role in the disease (11). Additionally, pediatric patients with NAFLD are more likely to have inflammation and fibrosis in the portal regions of the liver, rather than the typical pericentral distribution observed in adults (12, 13). This is clinically significant because periportal inflammation is associated with greater severity of liver disease (14). The factors that drive portal injury and rapid progression from steatosis to fibrosis in pediatric NASH are unclear (9). Furthermore, few studies have measured inflammation and fibrosis during the progression of NAFLD in pediatric patients. Notably, the innate and adaptive immune systems and antioxidant responses are relatively immature in the fetus (15–19), making the fetus more vulnerable to fuel overload and subsequent cellular damage. However, mechanistic studies to examine early development and progression of NAFLD using models that closely mimic the human condition are lacking.

Here, we leveraged our well-established nonhuman primate (NHP) model of chronic maternal Western-style diet (WSD) consumption to investigate the cellular mechanisms promoting fibrosis beginning in utero (20). We used second harmonic generation (SHG) imaging technology and RNAscope, alongside flow cytometry, to identify fibrosis, hepatic stellate cell (HSC) activation, and immune cell populations in livers from NHP fetuses exposed to maternal WSD compared with control diet (CD). We also measured the persistence of collagen deposition in postnatal NHP 1-year-old (1YO) offspring exposed to maternal WSD and investigated whether maternal dietary interventions could halt the development of NHP fetal hepatic fibrosis.

## Results

### Chronic maternal WSD consumption increased maternal obesity and insulin resistance as well as fetal hypoxemia and hepatic steatosis.

NHP females fed a chronic WSD develop obesity with increased body weight and percentage of body fat compared with females of similar age consuming a relatively isocaloric CD, as previously reported (20–23) (Table 1). WSD-fed dams also had increased insulin AUC during an i.v. glucose tolerance test compared with CD-fed females, as previously observed (22) (Table 1), supporting development of insulin resistance. Fetuses from mothers on WSD had a more than 2-fold increase in liver triglycerides compared with CD-exposed fetuses, as previously reported (20, 22) (Table 1). No differences in body weights between CD- and WSD-exposed fetuses were observed, as previously reported (22) (Table 1). Absolute liver weight was 8% higher in WSD-exposed compared with CD-exposed fetuses, yet liver weight relative to body weight was not different between the groups (Table 1). Umbilical artery serum pO_2_ levels were lower in the WSD group compared with the CD group, as previously reported (22) (Table 1).

### Fetuses exposed to maternal WSD had increased periportal collagen deposition.

**Figure 1:**
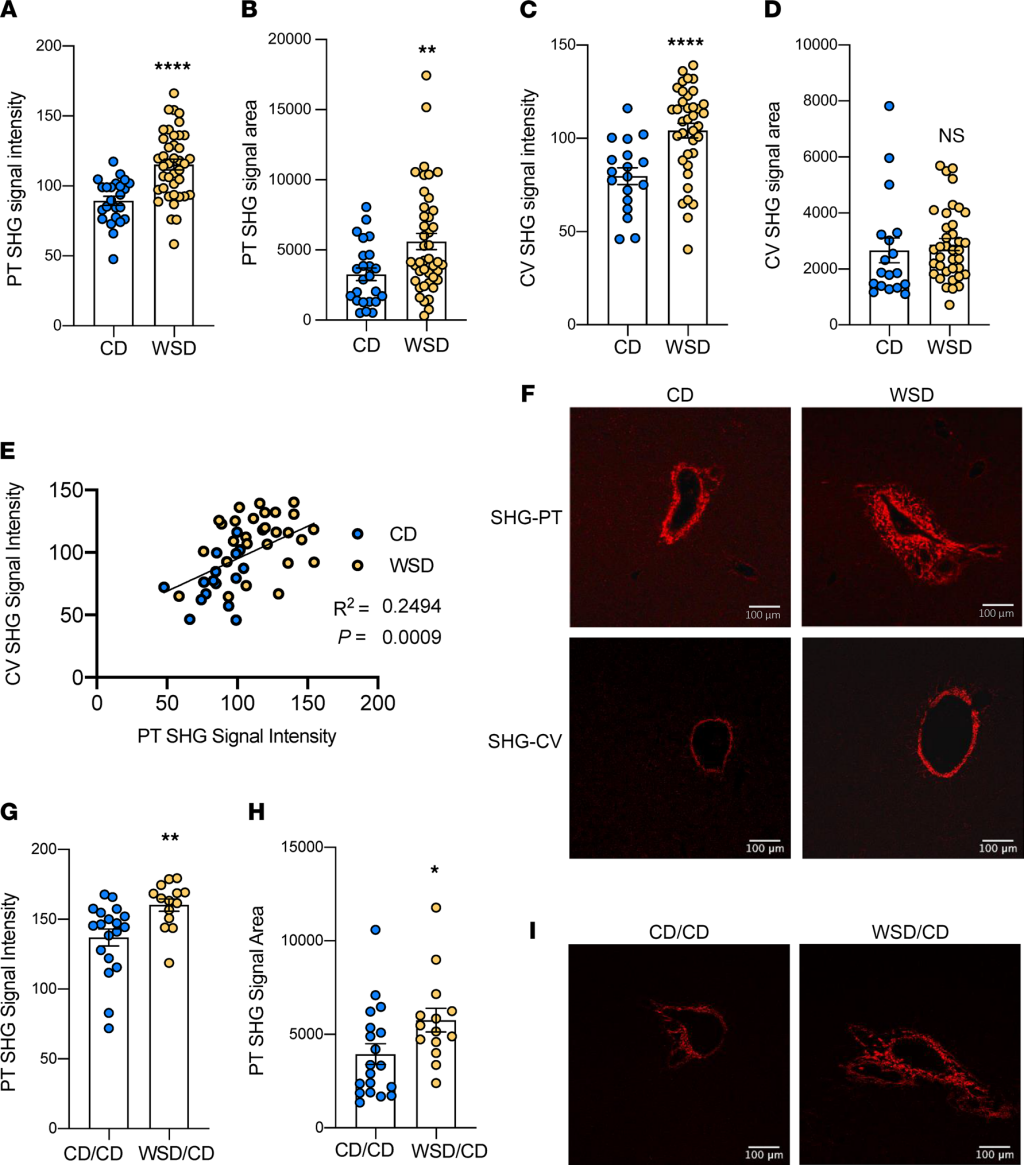
Maternal WSD exposure increases fibrosis in NHP fetal and 1YO offspring liver. SHG signal intensity (**A**) and signal area (**B**) of CD (blue) and WSD (yellow) portal triads (PT) in fetal livers; *n* = 24 C and *n* = 40 WSD PTs. SHG signal intensity (**C**) and signal area (**D**) of central veins (CV) in fetal livers; *n* = 18 CD and *n* = 37 WSD CVs. (**E**) Correlation between SHG signal intensity of PTs and SHG signal intensity of CVs in fetal livers. (**F**) Representative SHG images of PTs and CVs in fetal livers, with red indicating SHG signal. Scale bar: 100 μm. SHG signal intensity (**G**) and signal area (**H**) of 1YO offspring liver PTs; *n* = 19 CD/CD and *n* = 14 WSD/CD PTs. (**I**) Representative SHG images of PTs in CD/CD and WSD/CD 1YO livers, with red indicating SHG signal. Scale bar: 100 μm. Unpaired 2-tailed Student’s *t* test was used to test significance. *P < 0.05, **P < 0.005, ****P < 0.0005.

We used SHG/2-photon excitation fluorescence microscopy imaging, a highly sensitive technique used to detect collagen deposition in pediatric patients with NAFLD (24, 25), to quantify fibrillar collagen deposition directly without staining. WSD-exposed fetuses, compared with CD-exposed fetuses, had a 22% (*P* < 0.0005) increase in SHG signal intensity in the portal triad region of the liver (Figure 1, A and F) and a 43% (*P* < 0.005) increase in SHG signal area around the portal triads (Figure 1, B and F). SHG signal intensity around the central vein regions was increased by 28% (*P* < 0.0005) in livers from WSD-exposed fetuses compared with CD-exposed fetuses (Figure 1, C and F), yet the area of collagen around central veins was similar between the CD and WSD groups (Figure 1, D and F). SHG signal intensity in the portal triad and central vein regions was positively correlated (Figure 1E).

We next sought to determine if increased hepatic portal collagen deposition persisted in 1YO offspring from WSD-fed mothers after weaning onto a CD (WSD/CD). In WSD/CD-fed offspring, SHG signal intensity around the portal triads was increased by 14.5% (*P* < 0.005; Figure 1, G and I) and SHG area was increased by 31% (*P* < 0.05) compared with livers from CD/CD-fed offspring (Figure 1, H and I).

### Evidence for local HSC activation in portal triad regions of WSD-exposed fetal livers.

**Figure 2:**
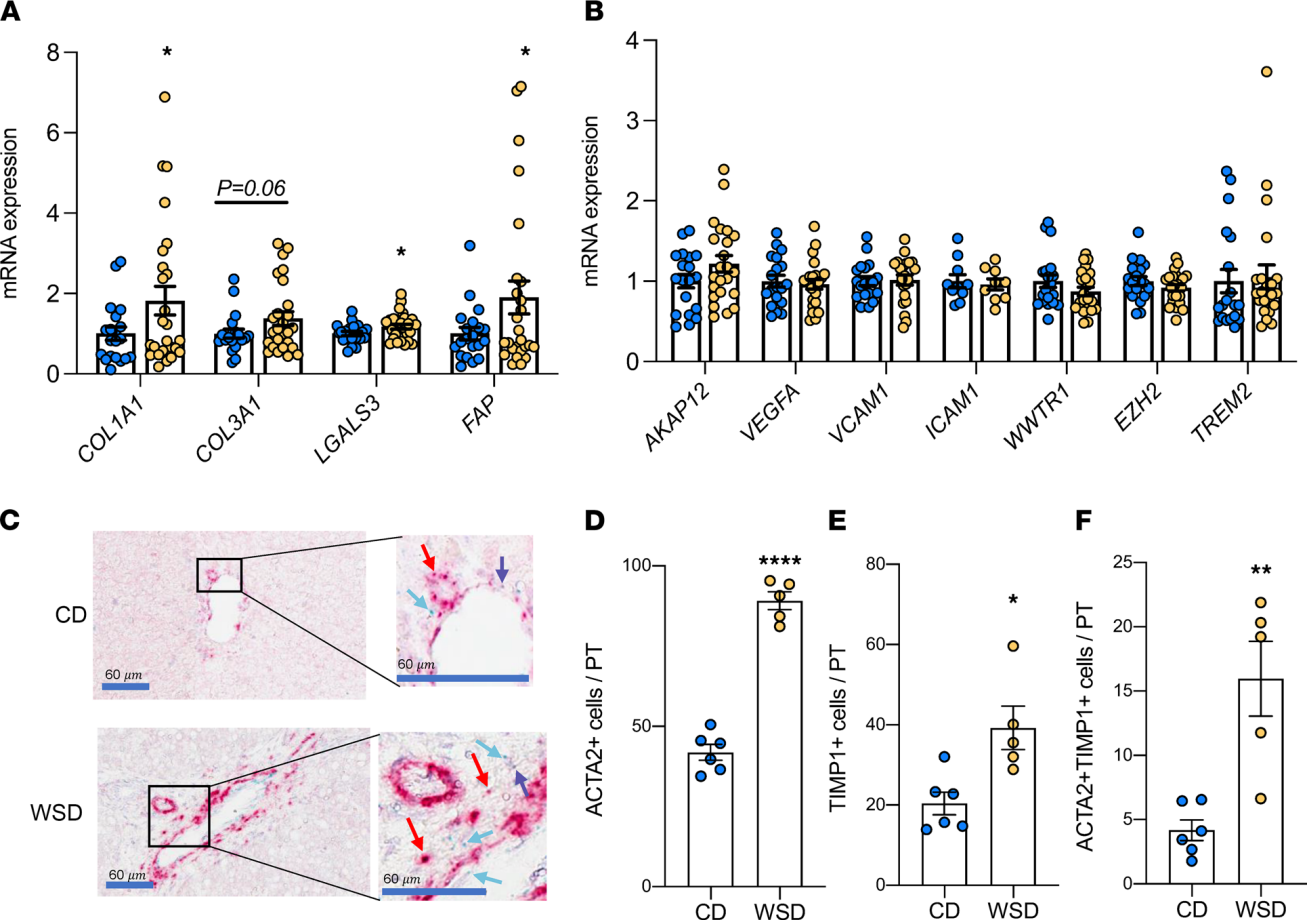
Evidence for increased collagen synthesis and stellate cell activation in NHP WSD-exposed fetal liver. Expression of genes in CD (blue) and WSD (yellow) fetal liver tissue associated with collagen synthesis and stellate cell activation (**A**). ANOVA with fixed effect for maternal diet is significant for all variables shown (*P* < 0.1). Individual post test comparisons were performed between CD and WSD; *n* = 20 CD and *n* = 26 WSD. *P < 0.05. (**B**) Expression of genes associated with endothelial dysfunction, the YAP/TAZ pathway, and myeloid cell inflammation; *n* = 20 CD and *n* = 26 WSD. (**C**) Representative images and zooms of portal triads of CD- and WSD-exposed fetal livers via RNAscope. Red arrows indicate *ACTA2*^+^ cells; blue arrows indicate *TIMP1*^+^ cells; purple arrows indicate *ACTA2* and *TIMP1* double-positive cells. Scale bar: 60 μm (boxed areas are enlarged and cropped on the right). Average numbers of *ACTA2*^+^ cells (**D**), *TIMP1*^+^ cells (**E**), and *ACTA2* and *TIMP1* double-positive cells (**F**) per portal triad (PT) via RNAscope; *n* = 6 CD and *n* = 5 WSD. Unpaired 2-tailed Student’s *t* test was used to test significance. *P < 0.05, **P < 0.005, ****P < 0.0005.

We next measured the expression of genes involved in collagen synthesis and HSC activation in livers from fetuses exposed to maternal CD or WSD. Expression of collagen synthesis genes *COL1A1* and *COL3A1* was widely variable but was increased (*COL1A1*) or tended to be increased (*COL3A1*, *P* = 0.06) in WSD-exposed livers (Figure 2A and Supplemental Table 1; supplemental material available online with this article; https://doi.org/10.1172/jci.insight.154093DS1). Expression of *LGALS3*, a marker of phagocytosis-induced HSC activation (26), and *FAP*, an HSC- and myofibroblast-derived marker of fibrosis (27), were also increased in WSD-exposed livers (Figure 2A and Supplemental Table 1). Expression of *ACTA2*, *TGFB1*, *PGDFA*, *PDGFRB*, and *TNFSF12*, additional genes expressed by activated HSCs and myofibroblasts, was not different between CD and WSD (Supplemental Table 1). No differences were detected in other biomarkers of canonical fibrosis development or inflammation: *AKAP12*, *VEGFA*, *VCAM1*, and *ICAM1* (well-known markers of endothelial activation); *WWTR1* and *EZH2* (key regulators of the YAP/TAZ [Hippo] pathway, ref. 28), or *TREM2* (a myeloid cell–derived antiinflammatory marker, ref. 29) (Figure 2B). Together, these data demonstrate variability in transcriptional activation of genes involved in collagen synthesis at the whole-liver level, without evidence for endothelial dysfunction or myeloid cell–derived antiinflammatory activation in WSD-exposed fetuses. Collagen synthesis and HSC activation genes were also not correlated with SHG signal area (Supplemental Figure 1A). Further, while WSD-fed dams were on the diet ranging from 1-9 years, SHG signal area in WSD-exposed fetuses was not increased in relation to length of maternal WSD consumption (Supplemental Figure 1B).

Given the histologic evidence for increased collagen localized to the portal triad regions, we used RNAscope with probes directed to *ACTA2* (30) and *TIMP1*, a marker of HSC activation induced by oxidative stress (31, 32), to localize stellate cell gene expression around the portal triad regions (Figure 2C). The number of *ACTA2*^+^ cells (*P* < 0.005) and *TIMP1*^+^ cells (*P* < 0.05) increased by approximately 2-fold in portal triad regions in WSD- compared with CD-exposed fetal livers (Figure 2, D and E, respectively). In addition, the number of cells that were positive for both *ACTA2* and *TIMP1* in the portal triad regions were 4-fold (*P* < 0.005) higher in WSD- compared with CD-exposed livers (Figure 2F). No difference in expression of S100A6, a marker of bile duct reaction (33, 34), was observed in CD- compared with WSD-exposed livers (Supplemental Figure 2).

### Shifts in immune cell populations in WSD-exposed fetal livers.

**Figure 3:**
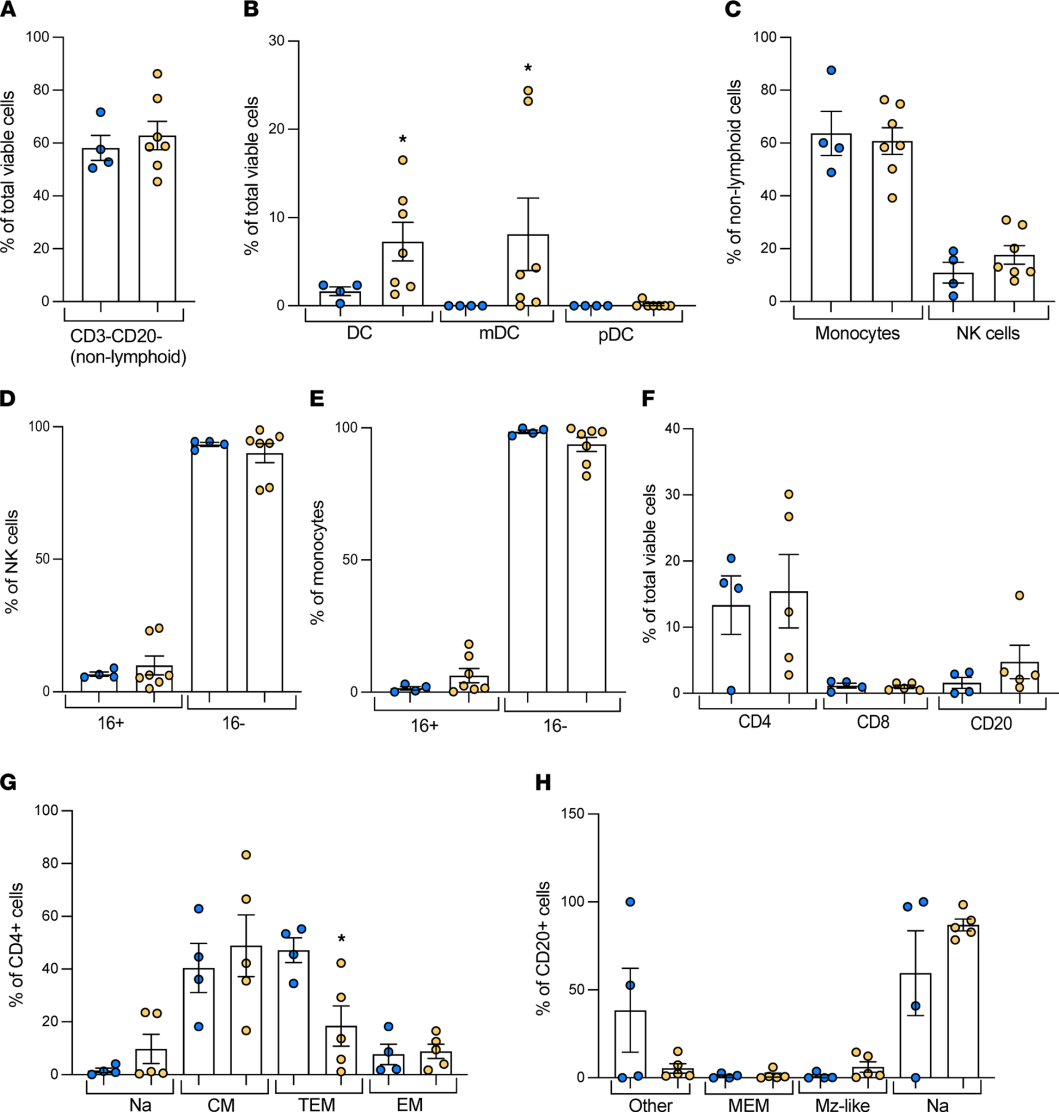
Flow cytometry of NHP fetal liver immune cells. Nonlymphoid myeloid cells and lymphoid cells in CD (blue) and WSD (yellow) fetal liver mononuclear cells, as a proportion of total viable cells or cell subsets by flow cytometry; *n* = 4 CD and *n* = 7 WSD. (**A**) Nonlymphoid cells, defined as CD3^–^CD20^–^, as a proportion of total viable cells. (**B**) DCs (DC), myeloid DCs (mDC), and plasmacytoid DCs (pDC) as a proportion of total viable cells. (**C**) Monocytes and natural killer (NK) cells as a proportion of nonlymphoid cells. NK cell subsets as a proportion of NK cells (**D**) and monocyte subsets as a proportion of monocytes (**E**) characterized by presence (16+) or absence (16-) of CD16. (**F**) CD4^+^ T cells, CD8^+^ T cells, and CD20^+^ B cells as a proportion of total viable cells. (**G**) CD4^+^ T cell subsets as a proportion of CD4^+^ T cells, corresponding to naive (Na), central memory (CM), transitional effector memory (TEM), and effector memory (EM) T cells. (**H**) CD20^+^ B cell subsets as a proportion of CD20^+^ B cells, corresponding to other (Other), memory (MEM), marginal zone–like (Mz-like), and naive B cells. Unpaired 2-tailed Student’s *t* test was used to test significance for all cell types other than mDCs, which had values of 0 in the CD group, so an unpaired 1-tailed *t* test was performed with Welch’s correction. *P < 0.05.

To investigate if specific immune cell populations were shifted in response to maternal WSD exposure in the fetal liver, we utilized flow cytometry with myeloid- and lymphoid-specific antibody panels to characterize these populations. In the myeloid panel, nonlymphoid cells (CD3^–^CD20^–^) were not different between groups (Figure 3A). WSD-exposed fetal livers had a nearly 4-fold increase in total DCs (CD3/CD20/CD14^–^HLA-DR^+^; *P* < 0.05) and a nearly 8-fold increase in myeloid DCs (mDC; CD11c^+^; *P* < 0.05) compared with CD-exposed livers, while plasmocytoid DCs (pDC; CD123^+^) were nearly absent in both groups (Figure 3B). No differences in the frequency of natural killer (NK; CD3/CD20/CD14^–^CD8a^+^) cells (Figure 3C) and their subsets (based on CD16; Figure 3D), or those of total monocytes (CD3/CD20^–^CD14^+^; Figure 3C) and their subsets (based on CD16; Figure 3E), were detected between CD and WSD groups. In the lymphoid panel, no differences in the frequency of CD4^-^ and CD8^+^ T cells or CD20^+^ B cells were detected (Figure 3F). However, a near 60% decrease (*P* < 0.05) in transitional effector memory (TEM; CD28^–^CD95^+^CCR7^+^) CD4^+^ T cells was found, in the absence of any changes in naive (CD28^+^CD95^–^CCR7^+^), central memory (CD28^+^CD95^+^CCR7^+^), or effector memory (CD28^–^CD95^+^CCR7^–^) CD4^+^ T cells (Figure 3G). No differences were found in CD20^+^ B cell subpopulations, including naive (CD27^–^IgD^+^), marginal zone–like (CD27^+^IgD^+^), memory (CD27^+^IgD^–^), or other memory (CD27^–^IgD^–^) B cells (Figure 3H). The frequency of CD8^+^ T cells was too small to examine naive and memory subsets (Figure 3F).

### Lack of systemic or hepatic inflammation in WSD-exposed fetuses.

**Figure 4:**
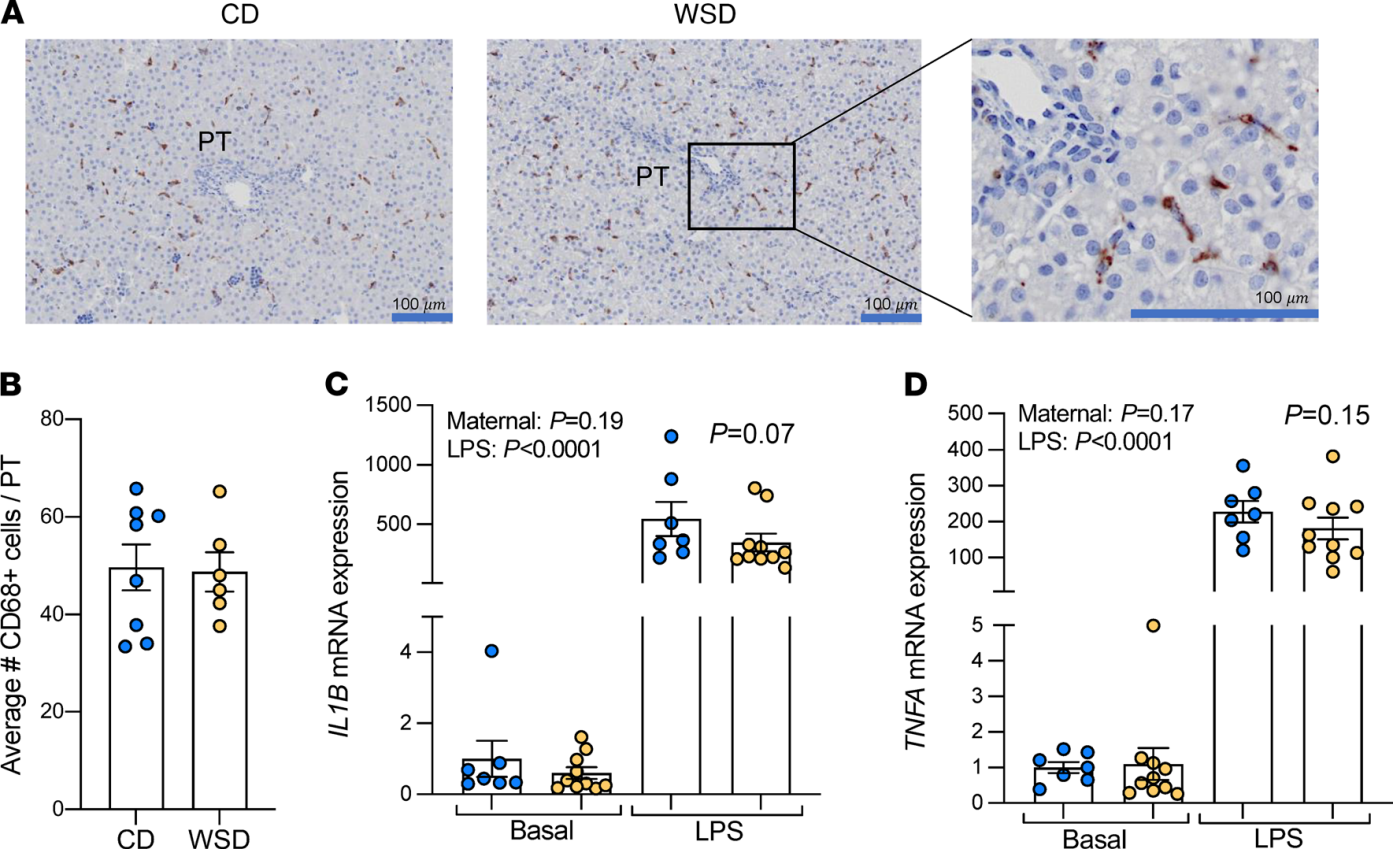
NHP fetal liver macrophage count and function. (**A**) Representative images of CD68-stained portal triad (PT) regions in CD- and WSD-exposed fetal liver tissue. Scale bar: 100 μm (boxed area is enlarged and cropped on the right). (**B**) CD68 staining quantification showing numbers of periportal CD68^+^ macrophages in CD (blue) and WSD (yellow) exposed fetal livers; *n* = 8 CD and *n* = 6 WSD. Unpaired 2-tailed Student’s *t* test was used to test significance. Liver macrophage gene expression of *IL1B* (**C**) and *TNF* (**D**) at baseline and in response to LPS; *n* = 7 CD and *n* = 10 WSD. A mixed model 2-way ANOVA was used to test effect of maternal diet and LPS treatment. Maternal effect and treatment effect *P* values are shown, and LPS x maternal diet interaction effect *P* values are shown above WSD bars.

Given the increase in DCs in WSD-exposed fetal livers, we further characterized inflammation in cells and tissues from fetal livers and fetal serum. The number of CD68^+^ macrophage cells around the portal triads (35) was not different between CD and WSD groups (Figure 4, A and B). We then isolated macrophages from WSD- and CD-exposed fetal livers and measured baseline gene expression and the response to LPS treatment. No differences were found in baseline gene expression of cytokines (*IL1B*, *TNF*, *MCP1*, *TLR4*, *CCR2*, *IL6*, *SOCS3*, *IL10*, *IL12B*, *TGFB1*, and *NFKB1A)* between CD- and WSD-exposed fetal macrophages (Figure 4, C and D, and Supplemental Figure 3). Treatment with LPS induced a robust inflammatory response (*P* < 0.05 for LPS treatment effect), with increased expression of *IL1B* and *TNF* (Figure 4, C and D, respectively), *MCP1*, *TLR4*, *IL6*, *SOCS3*, *IL12B*, and *NFKB1A* (Supplemental Figure 3) in both CD- and WSD-exposed liver macrophages, and no increases in *CCR2*, *IL10*, or *TGFB1* (*P* > 0.1 for treatment effect; Supplemental Figure 3). In response to LPS, *IL1B* (*P* = 0.07) and *TNF* (*P* = 0.15) gene expression was lower (37% and 34% decreased, respectively) in fetal liver macrophages from WSD- compared with CD-exposed fetuses (Figure 4, C and D, respectively). No differences in other inflammatory gene expression were found between CD- and WSD-exposed liver macrophages when stimulated with LPS (*MCP1*, *TLR4*, *CCR2*, *IL6*, *SOCS3*, *IL10*, *IL12B*, *TGFB1*, and *NFKB1A*; Supplemental Figure 3).

**Figure 5:**
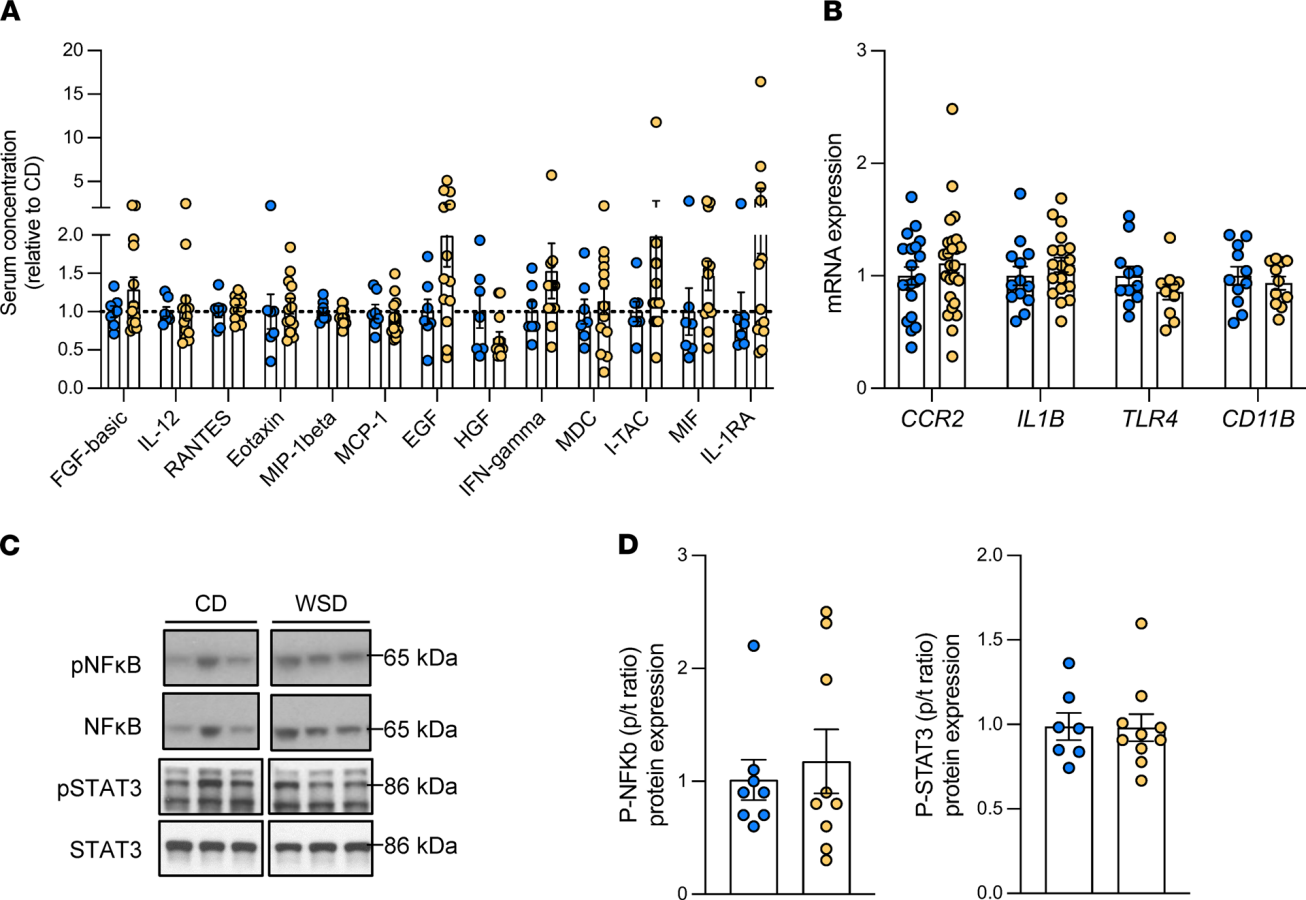
Systemic and hepatic cytokines and lack of inflammation in NHP WSD-exposed fetuses. (**A**) Cytokines, chemokines, and growth factors measured in paired umbilical vein and artery serum samples of CD (blue) and WSD (yellow) fetuses. Concentrations were averaged and expressed relative to mean in CD group; *n* = 6–7 CD and *n* = 13 WSD. (**B**) Liver tissue expression of inflammatory markers and hypoxia-related genes/targets; *n* = 11–20 CD and *n* = 11–28 WSD. Representative Western blots (**C**) and analysis (**D**) of the ratios of phosphorylated to total NFB and STAT3 protein expression in fetal livers (p/t ratio); *n* = 7–8 CD and *n* = 9–10 WSD. Unpaired 2-tailed Student’s *t* test was used to test significance.

We next measured proinflammatory cytokines, chemokines, and growth factors in fetal serum and gene expression of inflammatory markers in liver tissue. No differences were identified between vein and artery samples from the same fetus (Supplemental Table 2). Sixteen of 29 cytokines were below the limit of detection (IL-1β, IL-2, IL-4, IL-5, IL-6, IL-10, IL-15, IL-17, G-CSF, GM-CSF, IP-10, TNF-α, IL-8, MIG, MIP-1α, and VEGF). No differences in serum levels of FGF-basic, IL-12, RANTES, eotaxin, MIP-1β, MCP-1, EGF, HGF, IFN-γ, MDC, I-TAC, MIF, or IL-1RA were observed in the averaged vein and artery concentrations (Figure 5A). Similarly, no differences in expression of cytokines or immune response genes, including *CCR2*, *IL1B*, *TLR4*, and *CD11B*, in whole-liver tissue were observed (Figure 5B). In WSD-exposed livers, the ratio of phosphorylated (active) to total NF-κB or STAT3, both signaling proteins activated during inflammation (36, 37), was unchanged compared with CD-exposed livers (Figure 5, C and D). In addition, no differences in protein expression of IKKα, phosphorylated IKKα/β, phosphorylated IKKα/β/total IKKα ratio, and IκBα, all secondary NF-κB signaling proteins, were found between groups (Supplemental Figure 4). Together, these data suggest an absence of systemic or hepatic inflammation in fetuses exposed to maternal WSD- compared with CD-exposed fetuses.

### Maternal interventions prevented fetal collagen deposition and oxidative stress.

**Figure 6:**
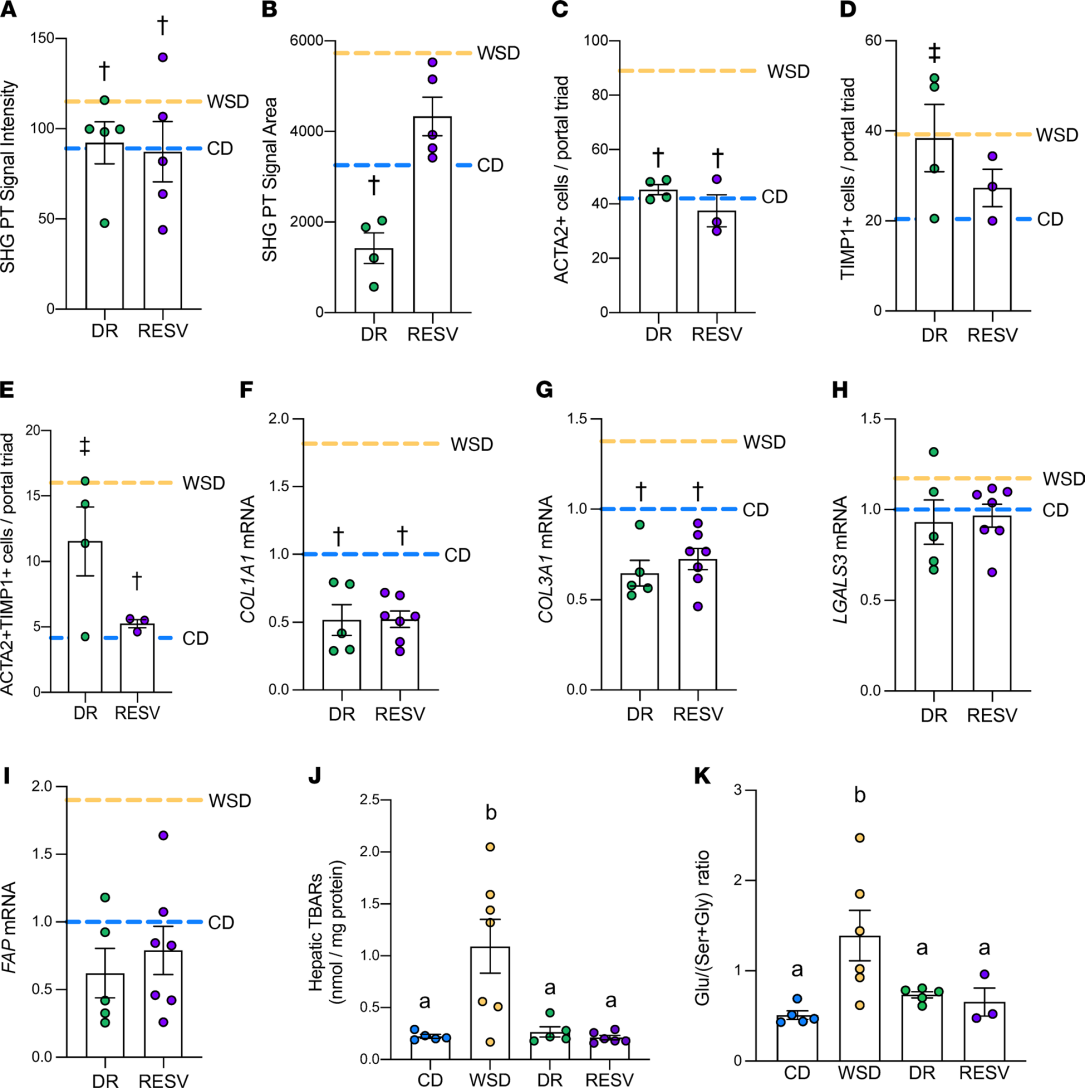
Effects of maternal diet interventions on NHP fetal hepatic collagen deposition. SHG signal intensity (**A**) and area (**B**) in portal triad regions from diet reversal (DR; green) and resveratrol (RESV; purple) fetal livers compared with mean of CD (blue dashed line) and WSD (yellow dashed line) groups; *n* = 4–5 DR and *n* = 5 RESV. RNAscope quantification of *ACTA2*^+^ (**C**) and *TIMP1*^+^ cells (**D**), and *ACTA2* and *TIMP1* double-positive cells (**E**) per portal triads in DR and RESV fetuses, compared with mean of CD (blue dashed line) and WSD (yellow dashed line) groups; *n* = 4 DR and *n* = 3 RESV. Expression of *COL1A1* (**F**), *COL3A1* (**G**), *LGALS3* (**H**), and *FAP* (**I**) in DR and RESV fetal liver tissue, compared with mean of CD (blue dashed line) and WSD (yellow dashed line) groups; *n* = 20 CD, *n* = 26 WSD, *n* = 5 DR, and *n* = 7 RESV. †*P* < 0.05 vs. WSD and NS vs. CD; ‡*P <* 0.05 vs. CD and NS vs. WSD (by ANOVA with fixed effect for maternal diet is shown and is significant for all variables shown (*P* < 0.05), unless noted otherwise. Individual post-test comparisons are indicated as different symbols). Unmarked bars indicate NS *P* values for CD and WSD comparison. (**J**) TBARS of CD (blue), WSD (yellow), DR, and RESV livers; *n* = 5 CD, *n* = 7 WSD, *n* = 5 DR, and *n* = 6 RESV. (**K**) Glutamate to serine + glycine (Glu/[Ser+Gly]) ratio of CD, WSD, DR, and RESV livers; *n* = 5 CD, *n* = 6 WSD, *n* = 5 DR, and *n* = 3 RESV. (**J** and **K**) ANOVA with fixed effect for maternal diet is shown and is significant for all variables shown (*P* < 0.05), unless noted otherwise. Individual post test comparisons are indicated as different letters. Bars with different symbols or letters represent groups with significant (*P* < 0.05) differences from one another, and bars sharing the same letter are not different from one another.

To determine if maternal diet interventions in the periconception period could prevent fetal hepatic fibrosis, we tested the effect of switching obese dams on the WSD to a CD approximately 2 months prior to conception (diet reversal [DR]) (22) and the effect of giving resveratrol supplementation to obese, WSD-fed females prior to and throughout pregnancy (RESV) (38) (Table 1). Collagen (SHG) signal intensity around the portal triad regions in DR and RESV fetuses was decreased (20% and 24%, respectively; *P* < 0.05 for both) compared with WSD-exposed fetuses and normalized to the CD group (Figure 6A and Supplemental Figure 5). SHG area was decreased in the DR group compared with the WSD group, but was not different in the RESV group compared with CD or WSD groups (Figure 6B and Supplemental Figure 5). We next sought to determine if reduced periportal collagen deposition was associated with reduced myofibroblast activation and reduced oxidative stress. The numbers of *ACTA2*^+^ cells were decreased in both DR and RESV livers (49% and 57%, respectively; *P* < 0.05; Figure 6C). In RESV livers, *TIMP1*^+^ cell counts were trending decreased (*P* < 0.15) compared with WSD-exposed livers and not different than CD-exposed livers (Figure 6D). DR livers had increased *TIMP1*^+^ cell counts compared with CD-exposed livers and were not significantly different than WSD-exposed livers (Figure 6D). The number of double-positive (*ACTA2* and *TIMP1*) cells was significantly decreased by 67% (*P* < 0.05) in RESV fetuses compared with WSD-exposed fetuses; however, the number of double-positive cells in the DR group remained increased compared with the CD group (Figure 6E).

Gene expression of *COL1A1* and *COL3A3* in DR and RESV fetal livers was decreased compared with that in WSD-exposed livers, whereas expression of HSC activation genes (*LGALS3*, *FAP*) was not different than in CD- or WSD-exposed livers, indicating partial resolution (Figure 6, F–I, and Supplemental Table 1). No difference in expression of *ACTA2* and *TGFB1* was observed in DR or RESV livers compared with CD- or WSD-exposed livers, but expression of *PDGFA* and *PDGFRB* in DR or RESV livers was significantly lower than in the CD or WSD groups, and expression of *TNFSF12* was significantly lower than the WSD group (Supplemental Table 1). WSD-exposed fetal livers had a 4-fold increase in TBARS, a marker of oxidative stress, compared with CD-exposed livers; in DR livers, TBARS decreased 76% compared with WSD-exposed livers (22) (Figure 6J). In RESV livers, fetal hepatic TBARS was decreased 80% (*P* < 0.05) relative to WSD-exposed livers (Figure 6J). The serum glutamate to serine + glycine ratio (Glu/[Ser+Gly]), a biomarker for fibrosis and oxidative stress in adult human NAFLD (39), was increased in the umbilical artery in WSD-exposed fetuses compared with CD-exposed fetuses, but was normalized in the DR and RESV groups (Figure 6K). Finally, fetal liver triglyceride levels were lower in the DR group, as previously reported (22), and in the RESV group (38), compared with the WSD group, and approached similar levels to the CD group (Table 1).

**Figure 7:**
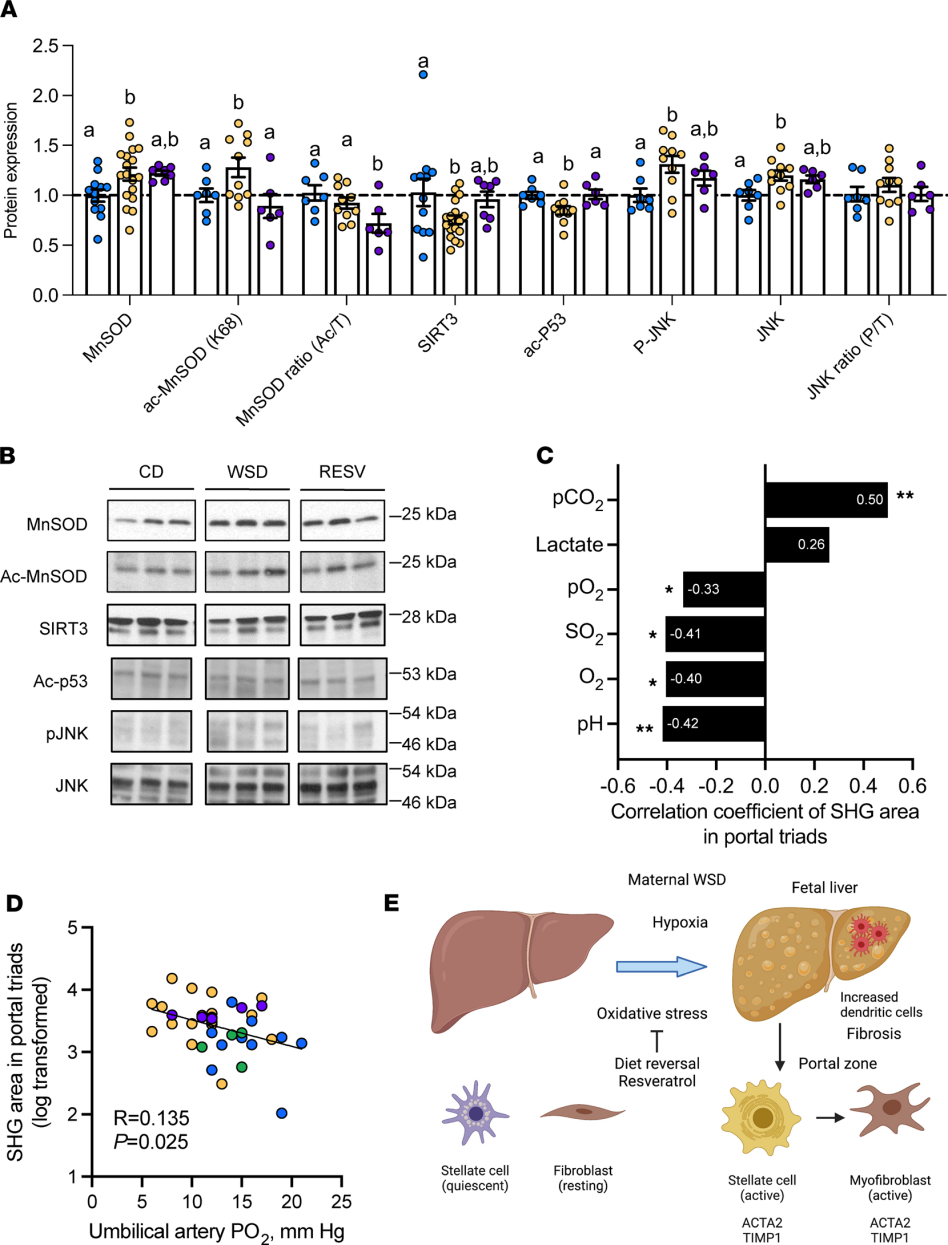
Effects of resveratrol on NHP fetal hepatic oxidative stress proteins. Quantitation (**A**) and representative Western blot images (**B**) of oxidative stress proteins. JNK and pJNK bands were identified and quantified within the 46–54 kDa range. *n* = 7–12 CD, *n* = 10–18 WSD, and *n* = 6–7 RESV. ANOVA with fixed effect for maternal diet is shown and is significant for all variables shown (*P* < 0.05), unless noted otherwise. Individual post test comparisons are indicated as different letters. Bars with different symbols or letters represent groups with significant (*P* < 0.05) differences from one another, and bars sharing the same letter are not different from one another. (**C**) Correlation coefficients for portal triad SHG signal area vs. umbilical artery cord blood measurements, using Pearson correlation coefficient to obtain the *P* values listed. *P < 0.05, **P < 0.005. (**D**) SHG area vs. umbilical artery pO_2_ measurements in CD (blue), WSD (yellow), DR (green), and RESV (purple) groups. (**E**) Maternal WSD increases hypoxia, fetal hepatic steatosis, oxidative stress and activation of hepatic stellate cells and myofibroblast formation in the portal zone. Dendritic cells are recruited in increased amounts to the fetal liver. The effects on liver steatosis and collagen formation were prevented by switching obese WSD-fed females to control diet prior to pregnancy and by supplementing obese WSD-fed females with resveratrol. Image created with BioRender.

Since resveratrol reduced oxidative stress and collagen deposition in the fetal liver even in the presence of WSD exposure, we further investigated oxidative stress pathways in livers of fetuses from RESV mothers compared with CD and WSD. Protein levels of the oxidative stress marker manganese superoxide dismutase (MnSOD) were increased by 21% (*P* < 0.05) in WSD-exposed livers compared with CD-exposed livers (Figure 7, A and B). Expression of acetylated MnSOD (Ac-MnSOD), the prooxidant form of MnSOD (40, 41), was also increased (*P* = 0.05; Figure 7, A and B). The ratio of acetylated/total MnSOD was not different between CD- and WSD-exposed livers (Figure 7A). In livers of RESV fetuses, Ac-MnSOD was decreased by 30% (*P* < 0.05) compared with WSD-exposed livers (Figure 7, A and B), yet total MnSOD protein abundance was not different. The ratio of acetylated/total MnSOD was decreased 22% compared with CD in RESV fetuses (Figure 7A). In addition, in WSD-exposed livers, protein expression of SIRT3, a deacetylase for MnSOD that promotes resolution of oxidative stress, was decreased by 27% (*P* < 0.05) (42, 43). SIRT3 protein expression in the RESV group was not different compared with CD- or WSD-exposed livers (Figure 7, A and B). Acetylated p53 (Ac-p53), another target of SIRT3 that mediates apoptosis and oxidative stress (44), was also decreased in the WSD-exposed fetal liver and improved to CD levels in RESV livers (Figure 7, A and B). Phosphorylated JNK (pJNK) protein expression was increased in WSD-exposed livers (20), and its expression in the RESV group was not different compared with the CD or WSD groups (Figure 7, A and B). Total JNK and the ratio of pJNK/total JNK was similar to levels of CD in RESV fetuses (Figure 7A).

### Factors associated with increased fetal liver SHG collagen deposition.

We next measured the association between SHG signal area and umbilical artery measurements that we previously reported in CD and WSD fetuses (22), including blood lactate concentrations, partial pressure of carbon dioxide (pCO_2_), partial pressure of oxygen (pO_2_), oxygen saturation (SO_2_), oxygen content (O_2_), and pH. SHG area was positively correlated with pCO_2_ and lactate and inversely associated with pH (Figure 7C). SHG area also was inversely correlated with pO_2_, SO_2_, and O_2_, which are measures of fetal oxygenation (Figure 7C). Importantly, umbilical arterial pO_2_ was increased in both the DR group, as previously reported (22), and the RESV group compared with the WSD group (Table 1). Further, umbilical arterial pO_2_ in CD, WSD, DR, and RESV fetuses was inversely correlated with SHG area (Figure 7D), supporting a relationship between hypoxemia and fibrosis.

## Discussion

Here, we demonstrate that maternal WSD exposure is associated with fibrillar collagen deposition and classic HSC and myofibroblast activation markers in the portal zone in NHP fetal liver. This pattern of collagen deposition occurred without hepatic or systemic inflammation and persisted in the periportal zone in maternal WSD-exposed offspring at 1 year of age. Our data further demonstrate that preventing WSD-induced oxidative stress in obese mothers either by diet switching or resveratrol treatment reduced collagen deposition and triglyceride content and attenuated markers of HSC activation in the fetal liver.

Clinically, children with zone 1 (periportal) distribution of fibrosis have an important subphenotype of pediatric NAFLD; they are characterized by increased periportal-based (vs. pericentral) inflammation relative to similarly affected adults (12, 45). SHG imaging is a highly sensitive technique that detects fibrillar collagens quantitatively, compared with more standard histological techniques, and has been efficacious in detecting subtle changes in periportal collagen deposition in pediatric NAFLD (24, 25, 46). SHG signal intensity represents the amount of collagen present; if the intensity is higher, it suggests that more collagen is present, possibly due to increased collagen density, while increased SHG area suggests that the extent of collagen spread is greater. Here, we found increased periportal fibrillar collagen intensity and area in the WSD-exposed fetal liver compared with CD-exposed livers. This pattern suggests that exposure to maternal WSD initiates spreading of collagen outward from the portal triads, which is associated with progression of NAFLD (8, 47). We also detected increased central vein SHG signal intensity, but not area, in the fetal liver, suggesting an increase in central vein collagen density. Overall, these observations suggest that exposure to maternal WSD increases collagen formation in discrete regions of the liver indicative of early fibrogenesis beginning in utero, much earlier than previously believed in the evolution of pediatric NAFLD (9, 48).

Upon liver injury, HSCs transform into myofibroblasts that express *ACTA2* (49). WSD-exposed fetuses had increased *ACTA2*^+^ and *TIMP1*^+^ cells in the portal triad regions, as well as increased *ACTA2* and *TIMP1* double-positive cells. *TIMP1* is induced and secreted by HSCs activated by macrophages and oxidative stress (31, 32, 50). In addition, *TIMP1* suppresses apoptosis of HSCs, which may lead to continued activation of HSCs to drive fibrosis (49, 51). While expression of collagen synthesis and HSC activation genes was significantly higher in whole-liver tissue from the WSD group compared with the CD group, we did not detect maternal WSD-driven increases in whole-liver expression of *ACTA2, TGFB1*, and other HSC activation-associated genes. This suggests that maternal WSD is associated with a highly localized pattern of collagen deposition that is difficult to detect when analyzing total liver RNA, because portal triad HSCs/myofibroblasts only represent a small portion of total liver cells. Interestingly, we found that switching WSD females to CD prior to and during pregnancy normalized SHG collagen intensity and area and expression of *ACTA2*, but not *TIMP1*, in DR fetal livers. This suggests that a subset of profibrogenic HSCs is sensitive to diet (52, 53), rather than a single homogenous cluster of activated HSCs, as described previously in mouse models of NASH (54–56). In another NHP model of maternal high-fat diet/obesity, baboon fetuses showed downregulation of Wnt-associated miR-199a-5p and miR-182-5p, a pattern associated with fibrogenesis (57, 58). Together, these results suggest that in utero exposure to maternal WSD is temporally and spatially associated with an increase in activation of profibrotic pathways in the fetus and may explain earlier-onset severe disease noted in some children with NASH (4, 9).

WSD-exposed fetuses showed an increase in classic markers of oxidative stress in the liver, including increases in TBARS content, 4-HNE labeling, and JNK activation (20, 22, 59). Lipid peroxidation products (such as 4-HNE) and free radicals produced by oxidative stress cause activation of HSCs and myofibroblasts (60, 61), which can lead to collagen deposition and fibrosis. Here, we demonstrated that supplementation of WSD-fed mothers with resveratrol reduced fetal hepatic oxidative stress, decreased periportal collagen deposition, and decreased *ACTA2*^+^ and *ACTA2* and *TIMP1* double-positive portal triad HSCs/myofibroblasts to levels comparable to CD fetuses. Activation of oxidative stress in WSD-exposed fetuses may result from decreased umbilical artery oxygenation (22) and impaired placental function (22, 62, 63). Importantly, we found that SHG area correlated positively with markers of fetal hypoxemia, suggesting that fetal stress and hypoxemia might be drivers of liver fibrosis in utero. Children who were born with low birth weight have a 2-fold increased risk for severe fibrosis (11), which may result from hypoxemia in association with growth restriction. Interestingly, experimental hypoxia-induced growth-restricted guinea pigs have increased fetal liver expression of *Tgfb1* and *Mmp2* as well as a trend for increased periportal collagen via trichrome staining (64). This suggests that fetal hypoxemia, in addition to lower antioxidant activity in the fetal liver (17–19), drives some aspects of early fibrosis development in WSD-exposed fetuses. Of note, resveratrol increases uterine artery blood flow in our model (38), likely increasing fetal O_2_ delivery, and crosses the placenta (38, 65, 66). This may confer a protective effect on fetal livers via direct effects in the liver or improved oxygen supply to the fetus. However, the therapeutic utility of resveratrol is limited by potential adverse effects in other developing fetal organs (38). As previously reported, maternal diet reversal reduced TBARS, improved fetal oxygenation, and normalized triglyceride levels (22); we show here that it normalized expression of collagen synthesis genes and a subset of HSC activation genes in whole liver and partially normalized RNA markers of HSC activation in portal triads. We note that SHG signal area in the RESV group is not different compared with that in the WSD group. However, both SHG signal area and SHG signal intensity were decreased in the DR group compared with the WSD group. Additionally, SHG area did not correlate with the length of time mothers were on the WSD. Collectively, this demonstrates that maternal diet, rather than obesity per se or length of time on WSD prior to pregnancy, is an important modifiable risk factor for fetal collagen deposition and steatosis.

One of the unique findings in the present study is the increase in DCs coupled with a decrease in TEM CD4^+^ T cells in the third trimester fetus in response to maternal WSD. This increase in DCs could be mediated by either increased recruitment into or production by the fetal liver in response to WSD exposure. DCs in healthy livers are largely immature and carry out tolerogenic responses to damage but change their function as liver damage progresses and may drive proinflammatory cytokine production and worsen liver damage in later stages of NAFLD (67). Despite an increase in DCs, we found no evidence for increased recruitment of CD68^+^ macrophages to the portal triad regions and no increase in inflammatory cytokines or NF-κB activation in WSD-exposed fetal liver or serum. Notably, inflammation is an adaptive response to tissue injury and a critical process for restoration of tissue functionality and homeostasis. During the third trimester, the primitive innate immune system, sourced by hematopoietic stem cells in the fetal liver, is in a developmental stage associated with a transition to postnatal hematopoiesis (68). Whether the persistence of fibrosis and lack of inflammation in utero is due to delayed maturation of the fetal innate immune system, lack of factors that stimulate production of inflammatory mediators, or lack of signals necessary for attracting macrophages to the site of liver injury, e.g., damage-associated molecular patterns, remains to be investigated. However, in livers from 1YO offspring of WSD-fed mothers with insulin resistance, we found an increase in macrophage recruitment and markers of inflammation, including *IL1B*, *CD68*, and *CD11B* in whole-liver tissue, and isolated hepatic macrophages were hyperresponsive to LPS (21). This suggests that, postnatally, macrophage recruitment and activation play a role in fibrosis development and liver inflammation in our model, similar to that observed in human NAFLD (6). These findings were observed in maternal WSD-exposed 1YO offspring regardless of postnatal diet, thus maternal WSD exposure may prime the offspring liver for inflammation later in life, potentially via oxidative stress in utero.

The increase in DCs coupled with a decrease in TEM CD4^+^ T cells could indicate an impaired ability to activate T cells in WSD-exposed fetal livers. CD4^+^ T cell expansion and activation, driven by DC-mediated antigen presentation, is key for initiating and worsening inflammation and fibrosis in NAFLD in some cases and stages of the disease, but evidence also exists for a role for CD4^+^ T cells in promoting resolution of inflammation and fibrosis (69, 70). Isolated liver macrophages from the WSD group demonstrated a blunted *IL1B* and *TNF* response to bacterial LPS, suggesting a functional dysregulation in fetal myeloid cells. These observations are consistent with those of a study of cord blood from babies born to obese mothers, showing dampened monocyte and DC response to TLR ligands and shifts in the proportions of CD4^+^ T cells (71). We speculate that this blunting of macrophage activity is a result of immune tolerance as a compensatory mechanism in response to challenges associated with WSD exposure. The net effect of these changes may set the stage for immune cell dysfunction and altered microbiome community structure and function postnatally (72–74). Future studies will identify if epigenetic reprogramming takes place in liver-derived macrophages that control inflammation, both in utero and postnatally.

Despite the robust link established between maternal obesity and offspring NAFLD, a key unanswered question is what is the nature of the primary molecular mechanism(s) driving the pathogenesis of the disease. Our results suggest that HSCs and myofibroblasts are sensitive to maternal WSD-associated oxidative stress in the fetal liver, which is spatially and temporally accompanied by periportal fibrosis, prior to emergence of inflammation (Figure 7E). The fetal liver receives approximately 80%–95% of oxygen- and nutrient-rich blood from the portal triad from the placenta via the umbilical vein, with the remainder supplied by the hepatic artery and little, if any, contribution from the portal vein, unlike in adults (75, 76). It is tempting to speculate that the unique blood supply to the portal triad during development, combined with factors produced by the WSD-exposed placenta (hypoxia, excess fatty acids), drives oxidative stress and HSC activation locally, making the region uniquely susceptible to liver injury that persists postnatally in pediatric NAFLD. Crucially, maternal diet intervention and resolution of fetal oxidative stress are capable of preventing these outcomes. This has important clinical implications, as maternal diet is a modifiable risk factor in women prone to obesity or excess weight gain, which could be leveraged to possibly prevent or delay onset of pediatric NAFLD and metabolic disease in the next generation. Further follow-up studies are underway in tissues and cells from juvenile NHP offspring to determine the molecular mechanisms unique to pediatric NAFLD progression.

## Methods

### Maternal WSD-induced obesity model.

Adult female Japanese macaques were fed WSD (36.6% calories from fat with 5.5% fructose and 8.8% sucrose) for 2–9 years prior to conception and throughout pregnancy to produce chronic WSD-induced obese mothers or maintained on CD (14.6% calories from fat with 2.8% sucrose and 0.2% fructose) as described previously (22, 77). Maternal body composition was measured by dual-energy X-ray absorptiometry (Hologic QDR Discovery A; Hologic Inc.) before pregnancy. Maternal body weight, plasma insulin, and glucose measurements were collected, and i.v. glucose tolerance tests were performed during the early third trimester of pregnancy (20, 38, 62). The data herein include singleton fetuses from CD- and WSD-fed mothers studied between 2008 and 2014 (23, 78). At approximately 130 days of gestation (gestation period is 165 days), fetuses were delivered by cesarean section while mothers were under anesthesia. Before cutting the umbilical cord, umbilical artery and venous blood samples were drawn from a section of cord that was clamped on each end (22). Umbilical artery serum samples were immediately analyzed for blood pO_2_, and venous and arterial serum was stored at –80°C for later analyses. Fetal weights were measured, and liver tissue samples were flash frozen in liquid nitrogen and processed for histology or stored at –80°C for subsequent analyses.

Two additional cohorts of fetal offspring were studied following maternal diet interventions. One cohort of obese females fed a chronic WSD for 7 years (*n* = 7) was placed on WSD supplemented with resveratrol to a final diet concentration of 0.37% (38) for 3 months prior to and throughout pregnancy. This produced maternal and fetal concentrations of resveratrol at 0.21–1.01 ng/mL and 0.91–1.93 ng/mL, respectively (38). Following cesarean section, these females were returned to WSD without resveratrol. Two years later, 5 of these same females were switched, as a group, to CD prior to the fall breeding season, allowed to breed naturally, and maintained on CD throughout pregnancy (22). The maternal phenotype was measured, and after cesarean section, fetal measurements and samples were collected as described earlier.

A cohort of offspring from CD- and WSD-fed mothers were born naturally and maintained with their mothers on their respective diets until weaning at approximately 6 months of age (21). At weaning, both CD- and WSD-fed offspring were placed on CD. At 1 year of age, the animals were sacrificed, and liver tissue samples were collected (21).

### Blood analyses.

Umbilical arterial (systemic fetal) blood levels of glutamate, serine, and glycine were measured in a previous metabolomic experiment (22) and are reported here as the ratio of glutamate to serine + glycine (Glu/[Ser+Gly]) (39). Fetal cytokines, chemokines, and growth factors were measured using a 29-plex Luminex NHP-specific cytokine panel (Thermo Fisher Scientific) using paired umbilical vein and artery serum samples collected at cesarean section. Serum levels were compared between vein and artery samples from the same fetus for each cytokine. The average of the vein and artery samples was calculated and used for each fetus and presented relative to the mean of the CD group for each cytokine.

### Liver histology and IHC analysis.

Liver tissue samples from the left lobe (fetal and 1YO offspring) were fixed in 10% zinc/formalin for 24 hours followed by storage in 70% ethanol. Samples were paraffin-embedded and 5 μm thick sections were prepared on slides. SHG signal was acquired from unstained paraffin slides using a Zeiss 780 LSM laser-scanning confocal/multiphoton-excitation fluorescence microscope with a 34-channel GaAsP QUASAR detection unit and nondescanned detectors for 2-photon fluorescence. The imaging settings were initially defined empirically with multiple samples to maximize the signal-to-noise ratio and to avoid signal saturation. These settings were kept constant for all measurements for comparative imaging and analysis. Six percent of a 2-photon Mai Tai laser (Spectra-Physics) tuned to 800 nm was used for excitation and emission signals corresponding to the autofluorescence and SHG signals, which were detected simultaneously through nondescanned detectors. Image processing was performed using Zeiss ZEN 2012 software. Eight to 12 fields of view (FOVs) containing 1–2 portal triads or 1–2 central veins were obtained for each liver sample using random blinded sampling, although portal triads were selected to be roughly similar sizes between the diet groups. Images were analyzed and quantified with ImageJ software (NIH) in order to obtain SHG signal intensity and area of SHG signal per FOV. The average values for all portal triad regions and central vein regions per animal were obtained.

For IHC detection of CD68^+^ or S100A6^+^ cells, slides were dehydrated with xylene and ethanol, and antigen retrieval was performed using citrate buffer (Advanced Cell Diagnostics [ACD]). Slides were immunolabeled with mouse CD68 (Agilent) or mouse S100A6 (Thermo Fisher Scientific) (Supplemental Table 3) followed by labeling with ImmPRESS anti-mouse IgG-HRP–conjugated secondaries antibody (Vector Laboratories). Staining was developed with DAB standard protocol (Vector Laboratories), counterstained with Hematoxylin QS (Vector Laboratories), and mounted with VectaMount solution (Vector Laboratories). Two sections per sample and 9–11 FOVs within 1 of the sections surrounding 1 portal triad each were analyzed. CD68^+^ cells were counted by investigators without knowledge of treatment group. S100A6 signal was quantified in the periportal regions with a custom-made Visiopharm application. Thresholding was applied to remove quantification of nonspecific background staining, and size filtering was performed to exclude all DAB^+^ signal with an area below 35 μm^2^ and above 1000 μm^2^ in order to quantify only bile duct cells expressing S100A6. The area of the signal of cells meeting these criteria was calculated and expressed relative to total tissue area.

### In situ mRNA hybridization.

Dual chromogenic RNAscope detection was used to perform in situ mRNA hybridization according the manufacturer’s protocol (ACD). Slides with 5 μm sections were deparaffinized in xylene, followed by rehydration in a series of ethanol/water washes. Following citrate buffer (ACD) antigen retrieval, slides were rinsed in deionized water and immediately treated with protease (ACD) at 40°C for 30 minutes in a HybEZII hybridization oven (ACD). Probes directed against α-smooth muscle actin (*ACTA2*) and TIMP metallopeptidase inhibitor 1 (*TIMP1*) mRNA and control probes were applied at 40°C in the following order: target probes, preamplifier, amplifier, and label probe. After each hybridization step, slides were washed 2 times at room temperature. mRNA chromogenic detection was performed followed by counterstaining with Hematoxylin QS (Vector Laboratories). Staining was visualized using an Aperio CS2 whole slide scanner with a ×40 objective (Leica Biosystems). Images were analyzed in ImageScope software (Leica Biosystems). Eight to 10 portal triads were imaged per sample, and *ACTA2*^+^, *TIMP1*^+^, and *ACTA2* and *TIMP1* double-positive cells were counted surrounding the portal triads.

### Fetal liver tissue analyses.

RNA was extracted from fetal liver (left lobe) samples, and gene expression was measured for collagen synthesis genes (*COL1A1*, *COL3A1*, *ACTA2*, *TGFB1*, *PGDFA*, *PDGFRB*, *TNFSF12*, *LGALS3*, *FAP*), markers of endothelial activation (*AKAP12*, *VEGFA*, *VCAM1*, *ICAM1*), YAP/TAZ activation (*WWTR1*, *EZH2*), myeloid cell antiinflammatory function (*TREM2*), and inflammation (*CCR2*, *IL1B*, *TLR4*, *CD11B*) using real-time qPCR (LightCycler 480, Roche) as described previously (21). The geometric mean of ribosomal protein S15 (*RPS15*), β-2 microglobulin (*B2M*), and hydroxymethylbilane synthase (*HMBS*) was used to normalize the data. See Supplemental Table 4 for primers. Data are presented relative to the mean of the CD group.

Whole cell lysates were prepared from fetal liver tissue (left lobe), and Western blotting was performed as previously described (79). Specificity of antibodies was verified by the presence of a single band at the expected molecular weight. Samples were run on 2 blots, with a loading control sample on each blot that was used to normalize data and compare data across blots. Representative blots and samples for each protein of interest are shown. Primary antibodies (Supplemental Table 3) at 1:500 dilution and appropriate secondary HRP-conjugated antibodies were used. Protein expression was quantified by densitometric analysis (22). Actin protein expression was measured to confirm equality of loading. Abundance of phosphorylated or acetylated proteins is expressed relative to total protein abundance. Data are presented relative to the mean of the CD group. TBARS and liver triglycerides were measured as previously described (22). CD, WSD, and DR liver triglycerides and TBARS have been previously published (22).

### Liver mononuclear cell isolation.

Liver mononuclear cells were obtained via perfusion and collagenase digestion of a portion of the right lobe of the liver from CD- and WSD-exposed fetuses (21). Briefly, hepatocytes were removed from the total mixture of digested cells by centrifugation at 100*g* for 5 minutes. The supernatant containing all nonparenchymal cells was filtered and spun at 800*g* for 10 minutes at 4°C to pellet nonparenchymal cells. Cells were resuspended in 24% Histodenz (MilliporeSigma), and gradients were prepared and spun at 1500*g* for 20 minutes at 4°C with brake turned off. Cells at the interface (referred to as liver mononuclear cells) were collected and washed, and aliquots were either cryopreserved in 90% FBS/10% DMSO, stored at –80°C, and used for flow analysis or used for isolating primary hepatic macrophages (described below).

### Characterization of liver immune cell proportions.

Cryopreserved liver mononuclear cells were thawed and immunolabeled with various antibodies and run on a flow cytometer (LSRII, BD Biosciences) to characterize liver immune cell proportions via fluorescence-activated cell sorting, with myeloid and lymphoid panels similar to published panels (80). 100,000 live cells were analyzed in each experiment. The myeloid panel included CD3, CD20, CD16, CD11c, CD14, CD123, CD8a, and HLA-DR and the lymphoid (T and B cell) panel included CD20, CD8b, CD4, CD27, CCR7, CD28, CD95, and IgD-biotin paired with Alexa Fluor 700 streptavidin (Supplemental Table 3). Subsets of CD8^+^ T cells were not reported due to a low overall cell count. Compensation controls were run with ultracomp ebeads (Thermo Fisher Scientific) during each run, and fluorescence minus one control was run for each fluorophore in the panel.

### Liver macrophages and LPS stimulation.

Liver mononuclear cells were plated on tissue culture plates in complete DMEM (with 2 mM L-glutamine, 1X pen/strep, 10 nM insulin, 100 nM dexamethasone, and 10% FBS) plus 55 μM 2-mercaptoethanol. After a 1- to 2-hour incubation, nonadherent cells were aspirated and media were replaced on the adherent cells, representing hepatic macrophages (81). Hepatic macrophages were incubated in complete media plus 2-mercaptoethanol for 24 hours. Hepatic macrophages were next incubated with serum-free media (DMEM with 2 mM L-glutamine, 1X pen/strep, and 0.1% BSA; basal) or LPS (100 ng/mL) in serum-free media for 3 hours. Cells were harvested for RNA isolation and qPCR analysis on *IL1B*, *TNF*, and *SOCS3*, as reported previously (21), as well as *MCP1* (*CCL2*), *TLR4*, *CCR2*, *IL6*, *IL10*, *IL12B*, *TGFB1*, and *NFKBIA*. To normalize expression data across multiple sets of cDNA synthesis and qPCR, the same 2 samples were run on each set and used to adjust data, and gene expression was normalized to *RPS15* and *CD11B* to control for presence of RNA from nonmacrophage cells. Primers are listed in Supplemental Table 4.

### Statistics.

Data were analyzed by 1-way ANOVA with fixed effect of maternal diet (CD, WSD, DR, RESV) or 2-way ANOVA with treatment and maternal diet effects using Prism (Graphpad software v. 9). Individual post test comparisons (Fisher’s LSD test) were made when the overall ANOVA *P* value was significant (*P* < 0.05 or *P* < 0.1 for 1-way ANOVA and *P* < 0.2 for 2-way ANOVA). For comparisons between CD and WSD groups alone, unpaired 2-tailed Student’s *t* test was used, and significance was determined as *P* < 0.05. When all values in the CD group were equal to 0 and variances were significantly (*P* < 0.05) different between groups, in comparisons with the WSD group, an unpaired 1-tailed *t* test was performed with Welch’s correction to account for inequality of variances. SHG imaging, RNAscope, liver immune cell populations, serum cytokine concentrations, liver macrophage gene expression, and liver protein expression, TBARS, and gene expression were measured in representative subsets of fetuses, as indicated in figure legends. Outliers were detected with Grubb’s test and were removed if *P* < 0.05.

### Study approval.

All animal procedures were conducted in accordance with the guidelines of the Institutional Animal Care and Use Committee of the Oregon National Primate Research Center and Oregon Health & Science University. The Oregon National Primate Research Center abides by the Animal Welfare Act and Regulations enforced by the United States Department of Agriculture.

## Author contributions

MJN, SRW, and JEF conceived and designed the study. MJN, SRW, and JEF wrote the manuscript, and MJN and RCJ created the figures and edited the manuscript. MJN and SRW performed the experiments, unless noted otherwise. MJN and ED performed SHG imaging and RNAscope analysis. SAN and SRW performed Western blotting experiments. IM performed flow cytometry. PK, JEF, SRW, KMA, CEM, and MG assisted with development of the NHP model and data interpretation.

## Supplementary Material

Supplemental data

## Figures and Tables

**Table 1:**
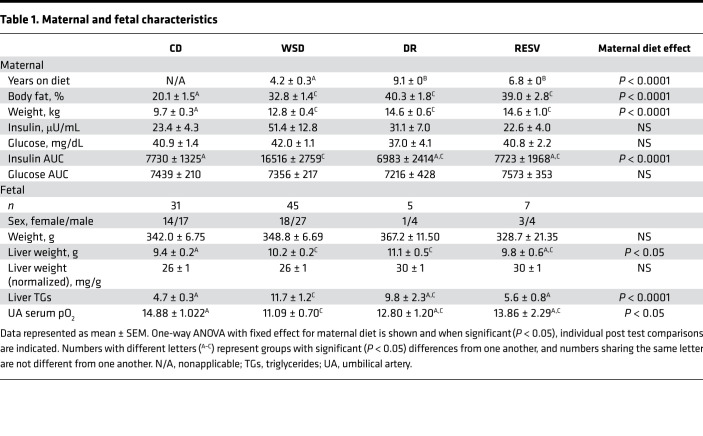
Maternal and fetal characteristics

## Version 1 12/22/2021

Electronic publication

## References

[1] Inoue Y (2018). Epidemiology of obesity in adults: latest trends. Curr Obes Rep.

[2] Carlson NS (2018). Antepartum care of women who are obese during pregnancy: systematic review of the current evidence. J Midwifery Womens Health.

[3] Godfrey KM (2017). Influence of maternal obesity on the long-term health of offspring. Lancet Diabetes Endocrinol.

[4] Mandala A (2020). Pediatric non-alcoholic fatty liver disease: nutritional origins and potential molecular mechanisms. Nutrients.

[5] Glastras SJ (2018). Maternal obesity increases the risk of metabolic disease and impacts renal health in offspring. Biosci Rep.

[6] Wesolowski SR (2017). Developmental origins of NAFLD: a womb with a clue. Nat Rev Gastroenterol Hepatol.

[7] Benedict M, Zhang X (2017). Non-alcoholic fatty liver disease: an expanded review. World J Hepatol.

[8] Pierantonelli I, Svegliati-Baroni G (2019). Nonalcoholic fatty liver disease: basic pathogenetic mechanisms in the progression from NAFLD to NASH. Transplantation.

[9] Goyal NP, Schwimmer JB (2016). The progression and natural history of pediatric nonalcoholic fatty liver disease. Clin Liver Dis.

[10] Cuzmar V (2020). Early obesity: risk factor for fatty liver disease. J Pediatr Gastroenterol Nutr.

[11] Newton KP (2017). Low and high birth weights are risk factors for nonalcoholic fatty liver disease in children. J Pediatr.

[12] Schwimmer JB (2005). Histopathology of pediatric nonalcoholic fatty liver disease. Hepatology.

[13] Crespo M (2016). Similarities and differences between pediatric and adult nonalcoholic fatty liver disease. Metabolism.

[14] Brunt EM (2009). Portal chronic inflammation in nonalcoholic fatty liver disease (NAFLD): a histologic marker of advanced NAFLD-clinicopathologic correlations from the nonalcoholic steatohepatitis clinical research network. Hepatology.

[15] Gale RP (1987). Development of the immune system in human fetal liver. Thymus.

[16] Popescu DM (2019). Decoding human fetal liver haematopoiesis. Nature.

[17] Elbarbry F, Alcorn J (2009). Ontogeny of glutathione and glutathione-related antioxidant enzymes in rat liver. Res Vet Sci.

[18] Frank L, Sosenko IR (1987). Prenatal development of lung antioxidant enzymes in four species. J Pediatr.

[19] Berkelhamer SK, Farrow KN (2014). Developmental regulation of antioxidant enzymes and their impact on neonatal lung disease. Antioxid Redox Signal.

[20] McCurdy CE (2009). Maternal high-fat diet triggers lipotoxicity in the fetal livers of nonhuman primates. J Clin Invest.

[21] Thorn SR (2014). Early life exposure to maternal insulin resistance has persistent effects on hepatic NAFLD in juvenile nonhuman primates. Diabetes.

[22] Wesolowski SR (2018). Switching obese mothers to a healthy diet improves fetal hypoxemia, hepatic metabolites, and lipotoxicity in non-human primates. Mol Metab.

[23] Elsakr JM (2021). Western-style diet consumption impairs maternal insulin sensitivity and glucose metabolism during pregnancy in a Japanese macaque model. Sci Rep.

[24] Liu F (2017). Second harmonic generation reveals subtle fibrosis differences in adult and pediatric nonalcoholic fatty liver disease. Am J Clin Pathol.

[25] Soon G, Wee A (2021). Updates in the quantitative assessment of liver fibrosis for nonalcoholic fatty liver disease: histological perspective. Clin Mol Hepatol.

[26] Jiang JX (2012). Galectin-3 modulates phagocytosis-induced stellate cell activation and liver fibrosis in vivo. Am J Physiol Gastrointest Liver Physiol.

[27] Levy MT (2002). Intrahepatic expression of the hepatic stellate cell marker fibroblast activation protein correlates with the degree of fibrosis in hepatitis C virus infection. Liver.

[28] Patel SH (2017). Hippo signaling in the liver regulates organ size, cell fate, and carcinogenesis. Gastroenterology.

[29] Ray K (2018). Liver: A protective role for TREM2 in liver injury. Nat Rev Gastroenterol Hepatol.

[30] Rockey DC (2013). Smooth muscle α actin (Acta2) and myofibroblast function during hepatic wound healing. PLoS One.

[31] Ghatak S (2011). Oxidative stress and hepatic stellate cell activation are key events in arsenic induced liver fibrosis in mice. Toxicol Appl Pharmacol.

[32] Nieto N (2006). Oxidative-stress and IL-6 mediate the fibrogenic effects of rodent Kupffer cells on stellate cells. Hepatology.

[33] Onsurathum S (2018). Proteomics detection of S100A6 in tumor tissue interstitial fluid and evaluation of its potential as a biomarker of cholangiocarcinoma. Tumour Biol.

[34] Dong XH (2021). S100 calcium binding protein A6 and associated long noncoding ribonucleic acids as biomarkers in the diagnosis and staging of primary biliary cholangitis. World J Gastroenterol.

[35] Gadd VL (2014). The portal inflammatory infiltrate and ductular reaction in human nonalcoholic fatty liver disease. Hepatology.

[36] Elsharkawy AM, Mann DA (2007). Nuclear factor-kappaB and the hepatic inflammation-fibrosis-cancer axis. Hepatology.

[37] He G, Karin M (2011). NF-κB and STAT3 — key players in liver inflammation and cancer. Cell Res.

[38] Roberts VH (2014). Beneficial and cautionary outcomes of resveratrol supplementation in pregnant nonhuman primates. FASEB J.

[39] Gaggini M (2018). Altered amino acid concentrations in NAFLD: impact of obesity and insulin resistance. Hepatology.

[40] Son Y (2011). Mitogen-activated protein kinases and reactive oxygen species: how can ROS activate MAPK pathways?. J Signal Transduct.

[41] Hjelmeland AB, Patel RP (2019). SOD2 acetylation and deacetylation: another tale of Jekyll and Hyde in cancer. Proc Natl Acad Sci U S A.

[42] Bause AS, Haigis MC (2013). SIRT3 regulation of mitochondrial oxidative stress. Exp Gerontol.

[43] Tao R (2014). Regulation of MnSOD enzymatic activity by Sirt3 connects the mitochondrial acetylome signaling networks to aging and carcinogenesis. Antioxid Redox Signal.

[44] Liu D, Xu Y (2011). p53, oxidative stress, and aging. Antioxid Redox Signal.

[45] Chalasani N (2008). Relationship of steatosis grade and zonal location to histological features of steatohepatitis in adult patients with non-alcoholic fatty liver disease. J Hepatol.

[46] Wanless IR (2020). Quantitative SHG-microscopy: unraveling the nano-architecture of the cirrhotic liver. Clin Res Hepatol Gastroenterol.

[47] Alkhouri N (2014). The development of the pediatric NAFLD fibrosis score (PNFS) to predict the presence of advanced fibrosis in children with nonalcoholic fatty liver disease. PLoS One.

[48] Feldstein AE (2009). The natural history of non-alcoholic fatty liver disease in children: a follow-up study for up to 20 years. Gut.

[49] Friedman SL (2008). Hepatic stellate cells: protean, multifunctional, and enigmatic cells of the liver. Physiol Rev.

[50] Lins MC (2005). Effects of maternal leptin treatment during lactation on the body weight and leptin resistance of adult offspring. Regul Pept.

[51] Minato Y (1983). The role of fat-storing cells in Disse space fibrogenesis in alcoholic liver disease. Hepatology.

[52] Rosenthal SB (2021). Heterogeneity of HSCs in a mouse model of NASH. Hepatology.

[53] Krenkel O (2019). Single cell RNA sequencing identifies subsets of hepatic stellate cells and myofibroblasts in liver fibrosis. Cells.

[54] Iwaisako K (2014). Origin of myofibroblasts in the fibrotic liver in mice. Proc Natl Acad Sci U S A.

[55] Mederacke I (2013). Fate tracing reveals hepatic stellate cells as dominant contributors to liver fibrosis independent of its aetiology. Nat Commun.

[56] Xiong X (2020). A single-cell perspective of the mammalian liver in health and disease. Hepatology.

[57] Murakami Y (2011). The progression of liver fibrosis is related with overexpression of the miR-199 and 200 families. PLoS One.

[58] Ceccarelli S (2013). Dual role of microRNAs in NAFLD. Int J Mol Sci.

[59] Grant WF (2011). Maternal high fat diet is associated with decreased plasma n-3 fatty acids and fetal hepatic apoptosis in nonhuman primates. PLoS One.

[60] Apte M (2002). Oxidative stress: does it ‘initiate’ hepatic stellate cell activation or only ‘perpetuate’ the process?. J Gastroenterol Hepatol.

[61] Gandhi CR (2012). Oxidative stress and hepatic stellate cells: a paradoxical relationship. Trends Cell Mol Biol.

[62] Salati JA (2019). Maternal high-fat diet reversal improves placental hemodynamics in a nonhuman primate model of diet-induced obesity. Int J Obes (Lond).

[63] Silvestro S (2020). Prenatal hypoxia and placental oxidative stress: insights from animal models to clinical evidences. Antioxidants (Basel).

[64] Sarr O (2016). The differential effects of low birth weight and Western diet consumption upon early life hepatic fibrosis development in guinea pig. J Physiol.

[65] Darby JRT (2019). Subcutaneous maternal resveratrol treatment increases uterine artery blood flow in the pregnant ewe and increases fetal but not cardiac growth. J Physiol.

[66] Bourque SL (2012). Maternal resveratrol treatment during pregnancy improves adverse fetal outcomes in a rat model of severe hypoxia. Placenta.

[67] Bernsmeier C, Albano E (2017). Liver dendritic cells and NAFLD evolution: a remaining open issue. J Hepatol.

[68] Gao X (2018). The hematopoietic stem cell niche: from embryo to adult. Development.

[69] Almeda-Valdes P (2015). The role of dendritic cells in fibrosis progression in nonalcoholic fatty liver disease. Biomed Res Int.

[70] Van Herck MA (2019). The differential roles of T cells in non-alcoholic fatty liver disease and obesity. Front Immunol.

[71] Sureshchandra S (2017). Maternal pregravid obesity remodels the DNA methylation landscape of cord blood monocytes disrupting their inflammatory program. J Immunol.

[72] Ma J (2014). High-fat maternal diet during pregnancy persistently alters the offspring microbiome in a primate model. Nat Commun.

[73] Pace RM (2018). Modulations in the offspring gut microbiome are refractory to postnatal synbiotic supplementation among juvenile primates. BMC Microbiol.

[74] Prince AL (2019). The development and ecology of the Japanese macaque gut microbiome from weaning to early adolescence in association with diet. Am J Primatol.

[75] Godfrey KM (2012). Fetal liver blood flow distribution: role in human developmental strategy to prioritize fat deposition versus brain development. PLoS One.

[76] Spurway J (2012). The development, structure and blood flow within the umbilical cord with particular reference to the venous system. Australas J Ultrasound Med.

[77] Thompson JR (2017). Exposure to a high-fat diet during early development programs behavior and impairs the central serotonergic system in juvenile non-human primates. Front Endocrinol (Lausanne).

[78] McCurdy CE (2016). Maternal obesity reduces oxidative capacity in fetal skeletal muscle of Japanese macaques. JCI Insight.

[79] Thorn SR (2009). Intrauterine growth restriction increases fetal hepatic gluconeogenic capacity and reduces messenger ribonucleic acid translation initiation and nutrient sensing in fetal liver and skeletal muscle. Endocrinology.

[80] Meyer C (2015). Flow cytometry-based methods to characterize immune senescence in nonhuman primates. Methods Mol Biol.

[81] Nnalue NA (1992). Salmonella choleraesuis and Salmonella typhimurium associated with liver cells after intravenous inoculation of rats are localized mainly in Kupffer cells and multiply intracellularly. Infect Immun.

