# Supplementary material for: Maternal Western diet exposure increases periportal fibrosis beginning in utero in nonhuman primate offspring
Source: JCI Insight. 2021 Dec 22;6(24):e154093. doi: 10.1172/jci.insight.154093 (PMC8783685; doi:10.1172/jci.insight.154093)
Supplement: Supplemental data [file jciinsight-6-154093-s228.pdf]

Maternal Western diet exposure increases periportal fibrosis beginning in utero in non-human primate offspring

Michael J. Nash,[1] Evgenia Dobrinskikh,[1] Sean A. Newsom,[1] Ilhem Messaoudi,[2] Rachel C. Janssen,[3] Kjersti M. Aagaard,[4] Carrie E. McCurdy,[5] Maureen Gannon,[6] Paul Kievit,[7] Jacob E. Friedman,[1,3] and Stephanie R. Wesolowski[1]

[1]Department of Pediatrics, Section of Neonatology, University of Colorado Anschutz Medical Campus, Aurora, CO, USA. [2]Department of Molecular Biology and Biochemistry, School of Biological Sciences, University of California Irvine, Irvine, CA, USA. [3]Harold Hamm Diabetes Center, University of Oklahoma Health Sciences Center, Oklahoma City, OK, USA. [4]Department of Obstetrics and Gynecology, Division of Maternal-Fetal Medicine, and Departments of Molecular and Human Genetics and Molecular and Cell Biology, Baylor College of Medicine, Houston, TX, USA. [5]Department of Human Physiology, University of Oregon, Eugene, OR, USA. [6]Division of Diabetes, Endocrinology, and Metabolism, Department of Medicine, Vanderbilt University Medical Center, Nashville, TN, USA. [7]Division of Cardiometabolic Health, Oregon National Primate Research Center, Oregon Health & Science University, Beaverton, OR, USA.

SAN's present address is: School of Biological and Population Health Sciences, Oregon State University, Corvallis, OR, USA.

Address correspondence to: Jacob E Friedman, Harold Hamm Diabetes Center, University of Oklahoma Health Sciences Center, 1000 N. Lincoln Blvd., Ste. 1200, Oklahoma City, OK 73104-3252, USA. Phone: 405.271.2824; Email: jed-friedman@OUHSC.edu.

Conflict of interest: The authors have declared that no conflict of interest exists.

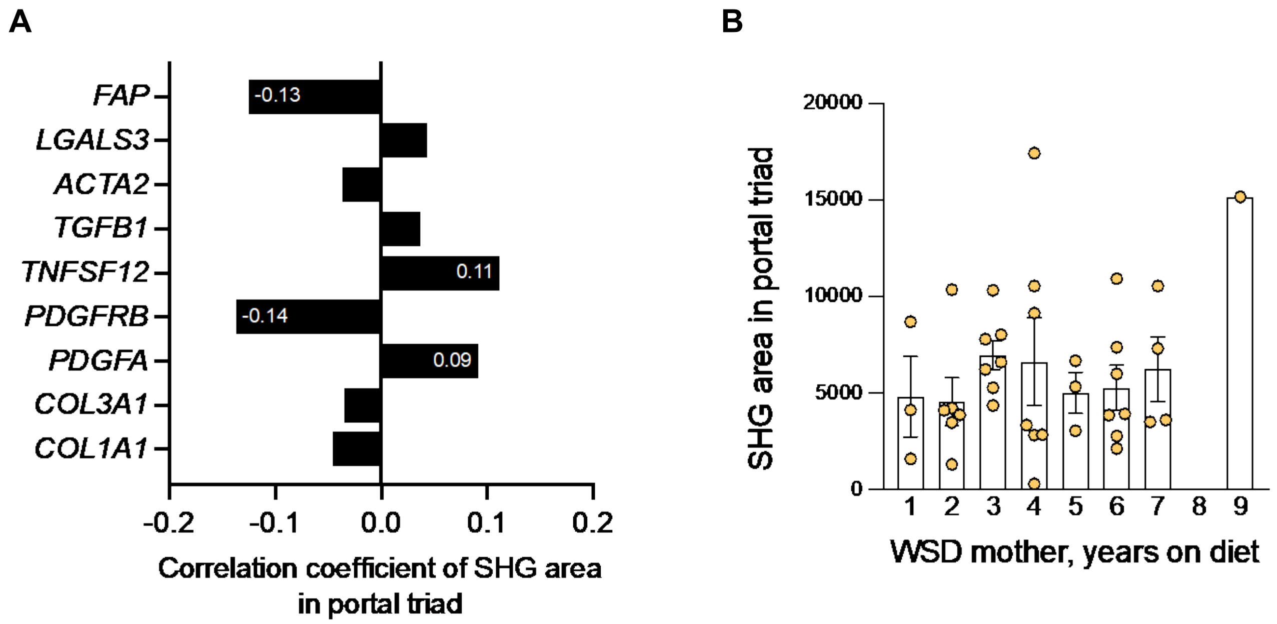

Supplemental Figure S1. Correlations of SHG area vs. liver gene expression, and SHG area by length of time WSD dams were on the diet. (**A**) Pearson's R values of SHG area with *FAP, LGALS3, ACTA2, TGFB1, TNFSF12, PDGFRB, PDGFA, COL3A1*, and *COL1A1; n* = 54 XY pairs, *n* = 19 CD; *n* = 35 WSD*;* all *P*>0.05. (**B**) SHG area organized by length of time (in years) the dams were on WSD.

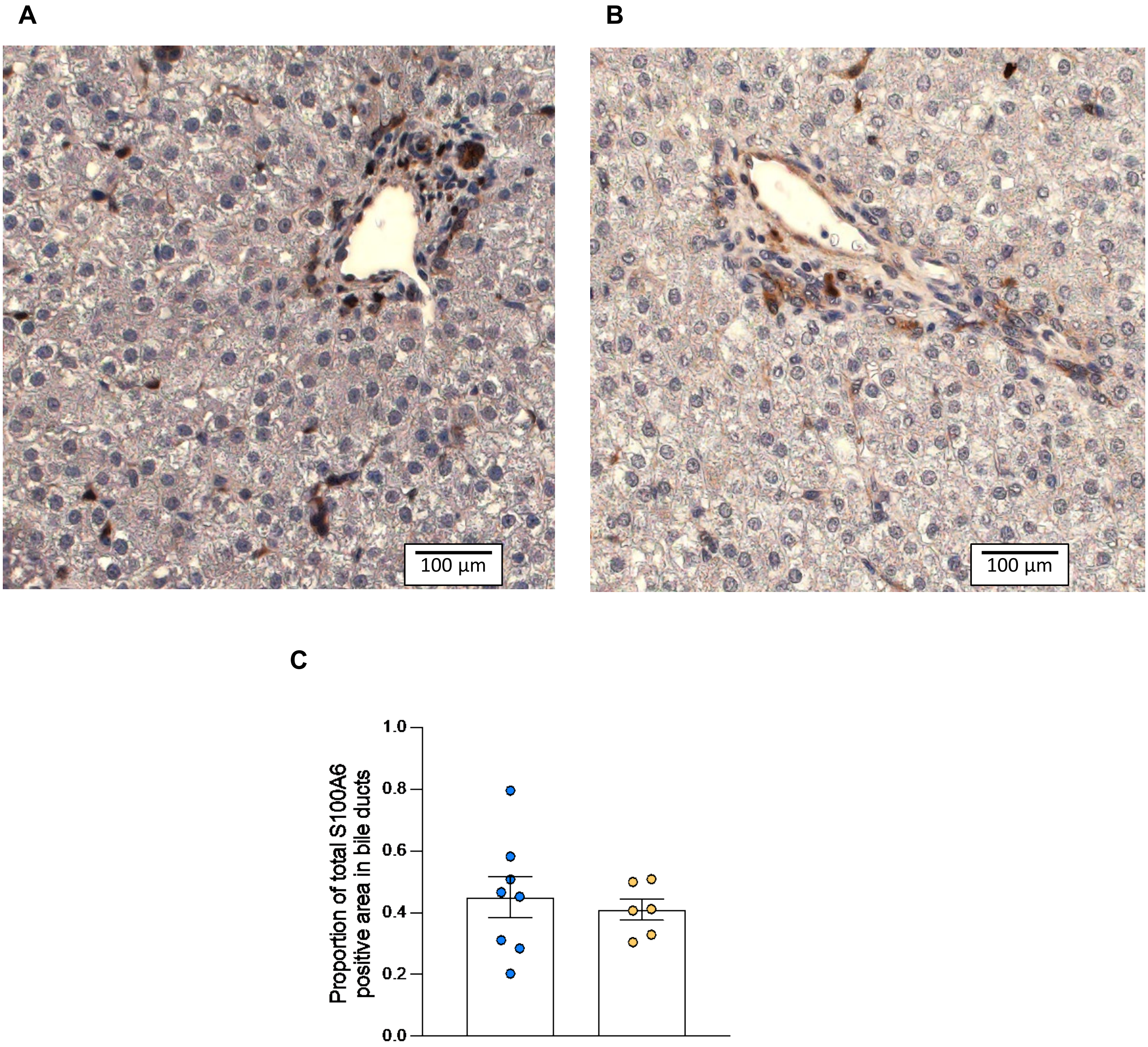

Supplemental Figure S2. Representative images and quantification of S100A6-stained fetal liver sections. Representative expression pattern of S100A6 in CD (**A**) and WSD (**B**) fetal livers. (**C**) Quantification of S100A6 staining in fetal liver bile ducts; *n* = 8 CD, *n* = 6 WSD.

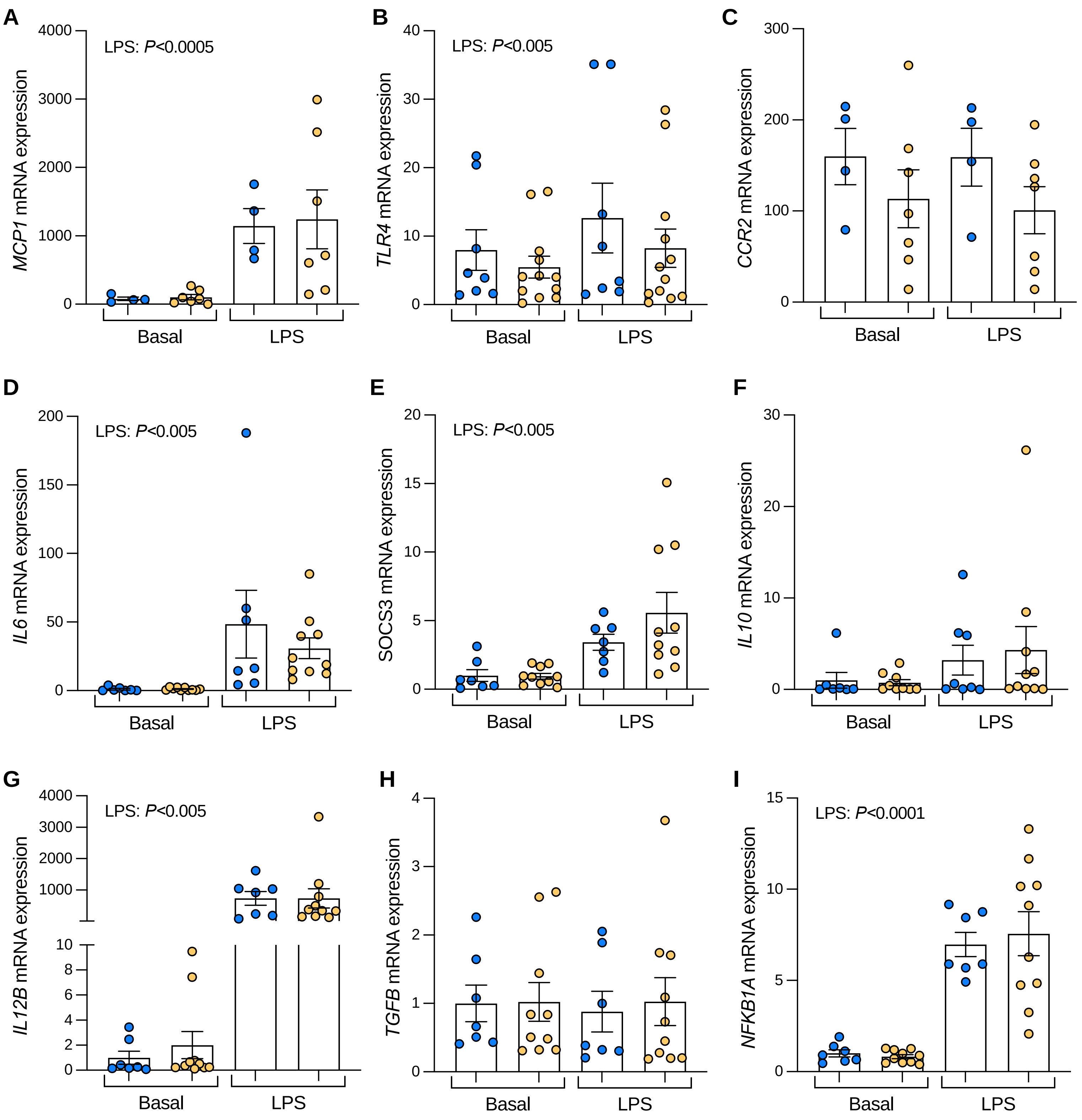

Supplemental Figure S3. Basal gene expression and LPS response of fetal liver macrophages. Basal expression and LPS-induced expression of *MCP1* (**A**), *TLR4* (**B**), *CCR2* (**C**), *IL6* (**D**), *SOCS3* (**E**), *IL10* (**F**), *IL12B* (**G**), *TGFB* (**H**), and *NFKB1A* (**I**). n = 4-7 CD, n = 7-10 WSD. A mixed model 2-way ANOVA was used to test effect of maternal diet and LPS treatment. Treatment effect P values are shown.

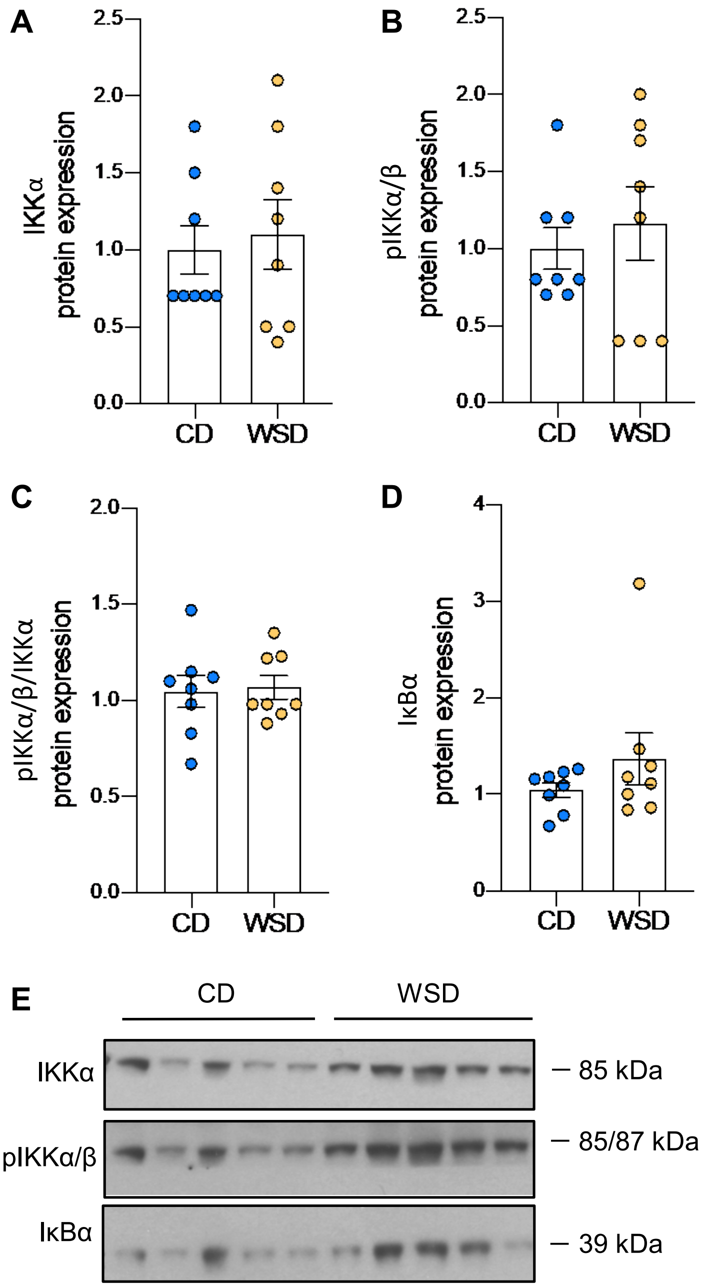

Supplemental Figure S4. Western blot data of NFκB signaling proteins. Fetal liver protein expression of IKKα (**A**), phosphorylated IKKα/β (pIKKα/β) (**B**), pIKKα/β /total IKKα ratio (**C**), and IκBα (**D**). (**E**) Representative Western blot images of IKKα, pIKKα/β, and IκBα. *n* = 8 CD and WSD.

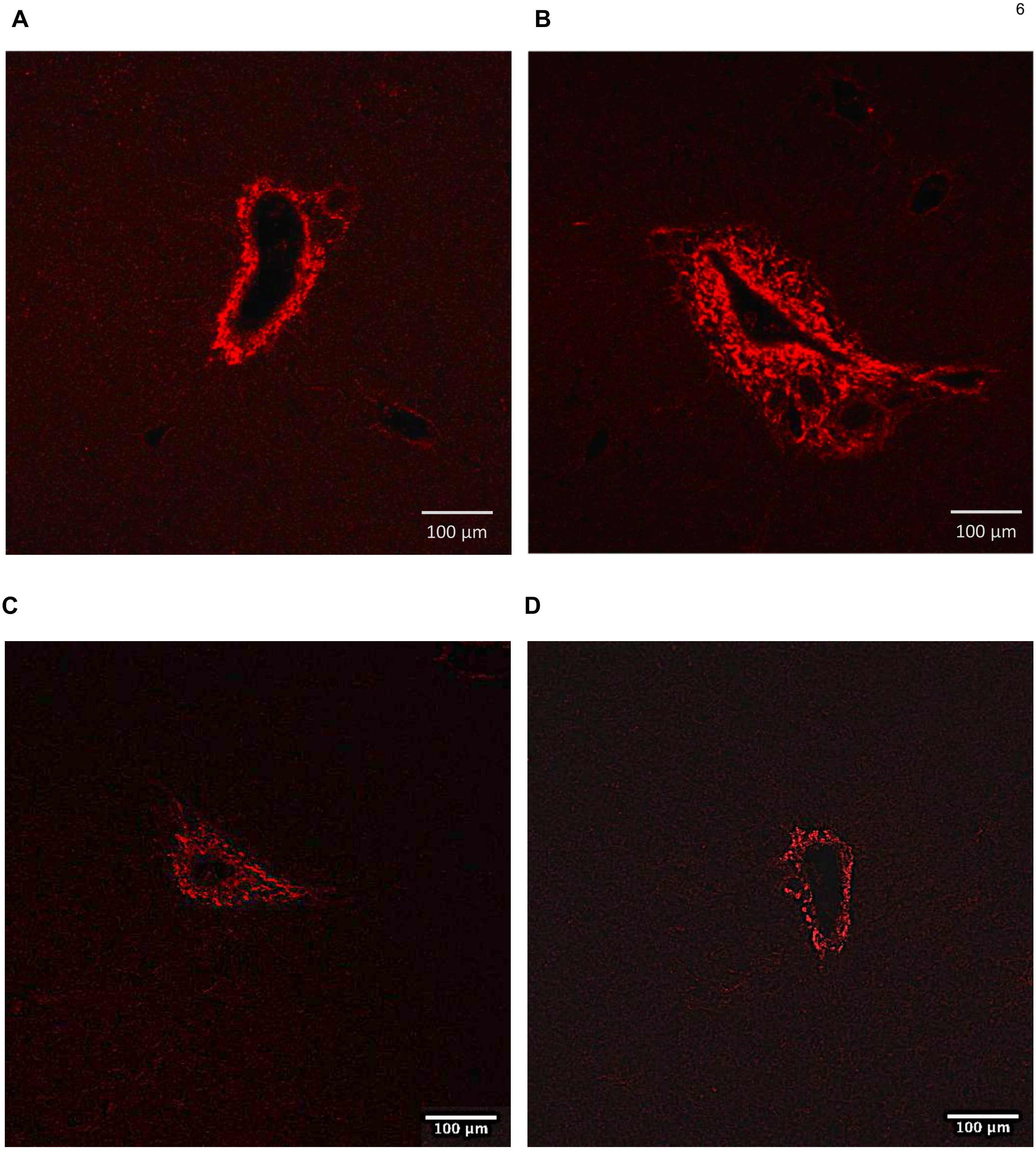

Supplemental Figure S5. SHG portal triad images from NHP fetal livers. Representative images of portal triad SHG signal from CD (**A**), WSD (**B**), DR (**C**). and RESV (**D**). CD and WSD images are reproduced from Fig. 1F for comparison.

Supplemental Table S1. Fetal liver collagen synthesis and HSC activation gene expression.

	CD	WSD	DR	RESV	Maternal diet effect
COL1A1	1.00 ± 0.16[a]	1.81 ± 0.35[b]	0.43 ± 0.11[a]	0.44 ± 0.06[a]	P<0.05
COL3A1	1.00 ± 0.11[a]	1.37 ± 0.17[a,b]	1.43 ± 0.07[a,c]	1.60 ± 0.06[a,c]	P<0.05
LGALS3	1.00 ± 0.05[a]	1.17 ± 0.06[b]	1.05 ± 0.12[a,b]	1.09 ± 0.06[a,b]	P=0.09
FAP	1.00 ± 0.15[a]	1.90 ± 0.41[b]	0.47 ± 0.18[a,b]	0.59 ± 0.18[a,b]	P=0.08
ACTA2	1.00 ± 0.13	1.18 ± 0.17	0.62 ± 0.18	0.44 ± 0.12	ns
TGFB1	1.00 ± 0.06	1.04 ± 0.05	0.67 ± 0.04	0.61 ± 0.04	ns
PDGFA	1.00 ± 0.07[a]	1.12 ± 0.08[a]	0.36 ± 0.03[b]	0.38 ± 0.06[b]	P<0.005
PDGFRB	1.00 ± 0.06[a]	1.00 ± 0.06[a]	0.74 ± 0.03[b]	0.76 ± 0.05[b]	P<0.05
TNFSF12	1.00 ± 0.07[a]	1.08 ± 0.08[a,b]	0.44 ± 0.04[a,c]	0.43 ± 0.06[a,c]	P<0.05

Data represented as mean ± SEM.
1-way ANOVA with fixed effect for maternal diet is shown and when significant (P < 0.1), individual post-test comparisons are indicated. Numbers with different letters represent groups with significant (P < 0.05) differences from one another, and numbers sharing the same letter are not different from one another.

Supplemental Table S2. NHP 29-plex cytokine panel results in fetal serum.

Animal ID	GROUP	Tube	FGF-basic	IL-1beta	G-CSF	IL-10	IL-6	IL-12	RANTES	Eotaxin
			pg/mL	pg/mL	pg/mL	pg/mL	pg/mL	pg/mL	pg/mL	pg/mL
31247	CD	umb artery	16.46	<4.33	<75.50	<4.18	<2.04	228.95	4,182.00	24.47
32183	CD	umb artery	14.41	<4.33	<75.50	<4.18	<2.04	305.79	3,967.00	18.38
32184	CD	umb artery	18.47	<4.33	<75.50	<4.18	<2.04	247.83	3,779.00	6.35
32231	CD	umb artery	16.46	<4.33	<75.50	<4.18	<2.04	266.95	3,285.00	15.72
32505	CD	umb artery	23.68	<3.38	<58.89	<3.26	<1.59	246.17	5,726.92	17.59
33312	CD	umb artery	10.18	<4.33	<75.50	<4.18	<2.04	210.34	3,033.00	57.16
33403	CD	umb artery	10.18	<4.33	<75.50	<4.18	<2.04	286.28	4,197.00	19.78
31926	CD	umb artery	16.46	<4.33	<75.50	<4.18	<2.04	174.02	3,690.00	38.94
33569	CD	umb artery	18.47	<4.33	<75.50	<4.18	<2.04	201.14	3,952.00	36.71
34212	CD	umb artery	10.18	<4.33	<75.50	<4.18	<2.04	266.95	4,585.00	32.29
34270	CD	umb artery	8.00	<4.33	<75.50	<4.18	<2.04	122.44	3,852.00	10.30
32085	WSD	umb artery	27.53	<3.81	<66.44	<3.68	<1.80	239.02	4,314.77	13.52
33078	WSD	umb artery	20.43	<4.33	<75.50	<4.18	<2.04	305.79	4,186.00	10.30
33074	WSD	umb artery	12.32	<4.33	<75.50	<4.18	<2.04	305.79	3,513.00	22.05
31003	WSD	umb artery	12.32	<4.33	<75.50	<4.18	<2.04	466.91	5,250.00	34.43
31146	WSD	umb artery	14.41	<4.33	<75.50	<4.18	<2.04	266.95	4,577.00	13.97
31280	WSD	umb artery	47.32	<4.33	<75.50	<4.18	<2.04	210.34	3,502.00	54.87
32204	WSD	umb artery	9.09	<4.33	<75.50	<4.18	<2.04	156.39	4,919.00	16.94
33101	WSD	umb artery	12.32	<4.33	<75.50	<4.18	<2.04	156.39	3,959.00	33.65
33138	WSD	umb artery	29.65	<4.33	<75.50	<4.18	<2.04	247.83	4,204.00	25.11
33263	WSD	umb artery	12.32	<4.33	<75.50	<4.18	<2.04	247.83	3,366.00	17.66
33314	WSD	umb artery	10.18	<4.33	<75.50	<4.18	<2.04	156.39	3,466.00	23.81
33334	WSD	umb artery	27.87	<4.33	<75.50	<4.18	<2.04	266.95	4,808.00	16.46
33351	WSD	umb artery	16.46	<4.33	<75.50	<4.18	<2.04	305.79	4,071.00	25.96
33498	WSD	umb artery	20.43	<4.33	<75.50	<4.18	<2.04	286.28	3,114.00	31.30
34292	WSD	umb artery	32.94	<3.90	<67.95	<3.76	<1.84	154.64	4,918.89	17.74
34293	WSD	umb artery	14.41	<4.33	<75.50	<4.18	<2.04	738.44	3,363.00	21.60
30959	WSD	umb artery	52.39	<4.33	<75.50	<4.18	<2.04	385.42	4,258.00	22.49
30871	WSD	umb artery	12.32	<4.33	<75.50	<4.18	<2.04	508.15	4,229.00	17.90
30968	WSD	umb artery	12.32	<4.33	<75.50	<4.18	<2.04	286.28	2,858.00	5.76
30969	WSD	umb artery	27.87	<4.33	<75.50	<4.18	<2.04	192.01	3,565.00	20.69
31247	CD	umb vein	15.44	<4.33	<75.50	<4.18	<2.04	247.83	4,251.00	21.60
32183	CD	umb vein	12.32	<4.33	<75.50	<4.18	<2.04	365.31	4,062.00	27.23
32184	CD	umb vein	12.32	<4.33	<75.50	<4.18	<2.04	247.83	4,654.00	9.75
32231	CD	umb vein	12.32	<4.33	<75.50	<4.18	<2.04	210.34	2,748.00	14.98
32505	CD	umb vein	14.41	<4.33	<75.50	<4.18	<2.04	266.95	5,238.00	15.48
33312	CD	umb vein	10.18	<4.33	<75.50	<4.18	<2.04	228.95	3,376.00	45.05
33403	CD	umb vein	13.37	<4.33	<75.50	<4.18	<2.04	345.33	4,329.00	26.81
31926	CD	umb vein	24.23	<4.33	<75.50	<4.18	<2.04	228.95	3,683.00	50.21

33569	CD	umb vein	12.32	<4.33	<75.50	<4.18	<2.04	192.01	4,695.00	28.47
34212	CD	umb vein	10.18	<4.33	<75.50	<4.18	<2.04	286.28	4,189.00	40.95
34270	CD	umb vein	10.18	<4.33	<75.50	<4.18	<2.04	106.24	4,219.00	10.84
32085	WSD	umb vein	14.41	<4.33	<75.50	<4.18	<2.04	286.28	3,630.00	5.76
33078	WSD	umb vein	12.32	<4.33	<75.50	<4.18	<2.04	238.36	4,108.00	9.20
33074	WSD	umb vein	12.32	<4.33	<75.50	<4.18	<2.04	286.28	4,748.00	13.97
31003	WSD	umb vein	14.41	<4.33	<75.50	<4.18	<2.04	528.87	4,516.00	29.49
31146	WSD	umb vein	16.46	<4.33	<75.50	<4.18	<2.04	345.33	5,269.00	16.46
31280	WSD	umb vein	16.46	<4.33	<75.50	<4.18	<2.04	192.01	3,725.00	29.29
32204	WSD	umb vein	12.32	<4.33	<75.50	<4.18	<2.04	192.01	4,477.00	11.37
33101	WSD	umb vein	12.32	<4.33	<75.50	<4.18	<2.04	174.02	4,269.00	36.33
33138	WSD	umb vein	26.07	<4.33	<75.50	<4.18	<2.04	210.34	4,104.00	28.88
33263	WSD	umb vein	10.18	<4.33	<75.50	<4.18	<2.04	305.79	4,032.00	20.69
33314	WSD	umb vein	12.32	<4.33	<75.50	<4.18	<2.04	156.39	4,686.00	23.16
33334	WSD	umb vein	37.24	<4.33	<75.50	<4.18	<2.04	286.28	5,629.00	16.94
33351	WSD	umb vein	12.32	<4.33	<75.50	<4.18	<2.04	238.36	4,963.00	22.49
33498	WSD	umb vein	18.47	<4.33	<75.50	<4.18	<2.04	210.34	3,592.00	29.69
34292	WSD	umb vein	20.43	<4.33	<75.50	<4.18	<2.04	228.95	4,134.00	22.05
34293	WSD	umb vein	10.18	<4.33	<75.50	<4.18	<2.04	570.47	3,163.00	18.85

IL-17	MIP-1alpha	GM-CSF	MIP-1beta	MCP-1	IL-15	EGF	IL-5	HGF	VEGF	IFN-gamma
pg/mL	pg/mL	pg/mL	pg/mL	pg/mL	pg/mL	pg/mL	pg/mL	pg/mL	pg/mL	pg/mL
<9.67	<11.42	<2.98	10.55	449.71	<13.67	33.41	<2.86	90.16	<0.46	7.84
<9.67	<11.42	<2.98	10.55	397.16	<13.67	10.83	<2.86	21.01	<0.46	5.03
<9.67	<11.42	<2.98	10.55	492.29	<13.67	16.99	<2.86	21.01	<0.46	<2.73
<9.67	<11.42	<2.98	10.55	498.25	<13.67	7.41	<2.86	<14.47	<0.46	<2.73
<7.54	<8.91	<2.32	13.53	300.51	<10.66	15.86	<2.23	54.83	<0.36	10.05
<9.67	<11.42	<2.98	10.55	706.34	<13.67	13.14	<2.86	<14.47	<0.46	<2.73
<9.67	<11.42	<2.98	10.55	568.15	<13.67	14.68	<2.86	21.01	<0.46	<2.73
<9.67	<11.42	<2.98	10.55	496.27	<13.67	39.80	<2.86	42.77	<0.46	5.03
<9.67	<11.42	<2.98	10.55	597.95	<13.67	22.33	<2.86	<14.47	<0.46	3.78
<9.67	<11.42	<2.98	<9.02	653.35	<13.67	7.03	<2.86	<14.47	<0.46	<2.73
<9.67	<11.42	<2.98	9.48	351.75	<13.67	6.66	<2.86	<14.47	<0.46	<2.73
<8.51	13.03	<2.62	14.55	328.59	<12.03	9.92	<2.52	48.60	0.83	8.91
<9.67	<11.42	<2.98	12.80	407.47	<13.67	20.43	<2.86	21.01	<0.46	5.03
<9.67	<11.42	<2.98	10.55	531.48	<13.67	21.57	<2.86	21.01	<0.46	7.84
<9.67	<11.42	<2.98	10.55	539.18	<13.67	31.60	<2.86	42.77	<0.46	7.84
<9.67	<11.42	<2.98	10.55	258.13	<13.67	25.15	<2.86	21.01	<0.46	7.84
<9.67	<11.42	<2.98	12.80	835.34	<13.67	132.93	<2.86	42.77	<0.46	5.03
<9.67	<11.42	<2.98	10.55	327.14	<13.67	13.91	<2.86	42.77	<0.46	7.84
<9.67	<11.42	<2.98	12.80	346.54	<13.67	21.95	<2.86	<14.47	<0.46	5.03
<9.67	<11.42	<2.98	15.15	438.79	<13.67	70.69	<2.86	21.01	<0.46	7.84
<9.67	<11.42	<2.98	<9.02	329.52	<13.67	9.30	<2.86	<14.47	<0.46	7.84
<9.67	<11.42	<2.98	10.55	440.88	<13.67	<1.01	<2.86	<14.47	<0.46	5.03
<9.67	<11.42	<2.98	10.55	367.17	<13.67	64.53	<2.86	<14.47	<0.46	5.03
<9.67	<11.42	<2.98	10.55	500.72	<13.67	32.33	<2.86	<14.47	<0.46	5.03
<9.67	<11.42	<2.98	10.55	469.17	<13.67	40.50	<2.86	<14.47	<0.46	5.03
<8.70	<10.28	<2.68	<8.12	340.64	<12.30	19.73	<2.57	<13.02	<0.41	<2.46
<9.67	<11.42	<2.98	10.55	649.77	<13.67	19.29	<2.86	<14.47	<0.46	29.42
<9.67	11.47	<2.98	12.80	696.29	<13.67	34.85	<2.86	42.77	0.78	5.03
<9.67	14.24	<2.98	12.80	733.67	50.02	7.41	<2.86	78.05	3.76	7.84
<9.67	<11.42	<2.98	10.55	244.69	<13.67	22.52	<2.86	21.01	<0.46	5.03
<9.67	14.24	<2.98	10.55	596.11	<13.67	38.92	<2.86	42.77	1.04	10.97
<9.67	<11.42	<2.98	10.55	497.76	<13.67	24.59	<2.86	42.77	<0.46	5.03
<9.67	<11.42	<2.98	15.15	433.03	<13.67	17.95	<2.86	<14.47	<0.46	<2.73
<9.67	<11.42	<2.98	10.55	478.78	<13.67	18.33	<2.86	42.77	<0.46	7.84
<9.67	<11.42	<2.98	17.60	399.88	<13.67	4.83	<2.86	21.01	<0.46	5.03
<9.67	<11.42	<2.98	10.55	356.35	<13.67	28.86	<2.86	42.77	<0.46	5.03
<9.67	<11.42	<2.98	<9.02	626.78	<13.67	14.49	<2.86	<14.47	<0.46	5.03
<9.67	<11.42	<2.98	10.55	714.62	<13.67	15.07	<2.86	66.07	<0.46	<2.73
<9.67	<11.42	<2.98	12.80	556.35	<13.67	56.91	<2.86	66.07	<0.46	7.84

<9.67	<11.42	<2.98	<9.02	589.63	<13.67	25.71	<2.86	21.01	<0.46	<2.73
<9.67	<11.42	<2.98	<9.02	701.10	<13.67	5.01	<2.86	<14.47	<0.46	5.03
<9.67	<11.42	<2.98	10.55	325.35	<13.67	7.78	<2.86	<14.47	<0.46	<2.73
<9.67	<11.42	<2.98	12.80	397.16	<13.67	4.83	<2.86	21.01	<0.46	7.84
<9.67	<11.42	<2.98	10.55	461.02	<13.67	17.76	<2.86	21.01	<0.46	5.03
<9.67	<11.42	<2.98	10.55	434.08	<13.67	19.48	<2.86	21.01	<0.46	7.84
<9.67	<11.42	<2.98	10.55	562.02	<13.67	37.52	<2.86	42.77	<0.46	7.84
<9.67	<11.42	<2.98	10.55	366.60	<13.67	21.95	<2.86	21.01	<0.46	5.03
<9.67	<11.42	<2.98	10.55	643.04	<13.67	39.63	<2.86	21.01	<0.46	10.97
<9.67	<11.42	<2.98	10.55	318.75	<13.67	15.45	<2.86	42.77	<0.46	5.03
<9.67	<11.42	<2.98	12.80	406.39	<13.67	17.18	<2.86	21.01	<0.46	5.03
<9.67	<11.42	<2.98	10.55	386.72	<13.67	58.20	<2.86	21.01	<0.46	5.03
<9.67	<11.42	<2.98	10.55	404.77	<13.67	4.30	<2.86	21.01	<0.46	5.03
<9.67	<11.42	<2.98	12.80	628.59	<13.67	15.84	<2.86	21.01	<0.46	5.03
<9.67	<11.42	<2.98	10.55	345.96	<13.67	70.08	<2.86	21.01	<0.46	5.03
<9.67	<11.42	<2.98	12.80	417.67	<13.67	26.09	<2.86	<14.47	<0.46	5.03
<9.67	<11.42	<2.98	10.55	444.52	<13.67	36.99	<2.86	21.01	<0.46	<2.73
<9.67	<11.42	<2.98	<9.02	432.50	<13.67	28.49	<2.86	21.01	<0.46	<2.73
<9.67	<11.42	<2.98	<9.02	605.78	<13.67	<1.01	<2.86	<14.47	<0.46	25.48

MDC	I-TAC	MIF	IL-1RA	TNF-alpha	IL-2	IP-10	MIG	IL-4	IL-8
pg/mL	pg/mL	pg/mL	pg/mL	pg/mL	pg/mL	pg/mL	pg/mL	pg/mL	pg/mL
752.42	48.08	>3,860.00	32.35	<5.11	<5.67	<0.66	<3.11	<20.11	4.77
1,262.00	14.06	428.21	52.14	<5.11	<5.67	<0.66	<3.11	<20.11	5.33
545.12	24.73	537.19	36.73	<5.11	<5.67	<0.66	<3.11	<20.11	7.74
366.13	<9.33	581.07	69.53	<5.11	<5.67	<0.66	<3.11	<20.11	4.77
643.01	24.69	851.90	49.91	<3.99	<4.42	<0.51	<3.99	<15.69	<3.52
1,328.00	24.73	1,413.00	45.54	<5.11	<5.67	<0.66	<3.11	<20.11	18.23
830.95	24.73	1,010.00	130.87	<5.11	<5.67	<0.66	<3.11	<20.11	<4.51
630.07	14.06	2,812.00	49.94	<5.11	<5.67	<0.66	<3.11	<20.11	5.91
1,425.00	24.73	657.64	45.54	<5.11	<5.67	<0.66	<3.11	<20.11	<4.51
1,935.00	14.06	629.56	45.54	<5.11	<5.67	<0.66	<3.11	<20.11	9.69
1,194.00	<9.33	1,131.00	19.50	<5.11	<5.67	<0.66	<3.11	<20.11	<4.51
362.93	41.11	1,915.91	49.25	<4.50	<5.00	<0.58	7.23	<17.70	12.53
671.50	24.73	1,200.00	41.14	<5.11	<5.67	<0.66	6.36	<20.11	<4.51
457.21	24.73	1,408.00	260.98	<5.11	<5.67	<0.66	<3.11	<20.11	<4.51
1,425.00	36.18	846.61	156.48	<5.11	<5.67	<0.66	<3.11	<20.11	<4.51
319.38	36.18	1,957.00	60.88	<5.11	21.12	0.72	<3.11	<20.11	<4.51
1,393.00	24.73	>3,860.00	107.10	<5.11	<5.67	<0.66	<3.11	<20.11	5.91
366.13	24.73	641.70	386.27	<5.11	<5.67	<0.66	<3.11	<20.11	<4.51
671.50	24.73	1,142.00	21.59	<5.11	<5.67	<0.66	<3.11	<20.11	<4.51
752.42	48.08	2,343.00	148.07	<5.11	<5.67	<0.66	<3.11	<20.11	5.33
1,733.00	36.18	907.01	756.82	<5.11	<5.67	<0.66	<3.11	<20.11	<4.51
752.42	24.73	883.73	52.14	<5.11	<5.67	<0.66	<3.11	<20.11	<4.51
1,393.00	24.73	3,353.00	34.54	<5.11	<5.67	<0.66	<3.11	<20.11	6.51
630.07	14.06	2,214.00	58.70	<5.11	<5.67	<0.66	<3.11	<20.11	<4.51
1,762.00	24.73	2,709.00	45.54	<5.11	<5.67	<0.66	<3.11	<20.11	<4.51
194.32	<8.40	1,804.44	57.93	<4.60	<5.10	<0.59	<2.80	<18.10	<4.06
1,295.00	366.45	2,212.00	247.31	<5.11	<5.67	29.80	6.36	<20.11	<4.51
671.50	60.22	>3,860.00	214.12	<5.11	<5.67	2.13	12.96	<20.11	7.74
366.13	238.48	1,710.00	109.12	<5.11	21.12	5.04	12.96	<20.11	<4.51
1,125.00	14.06	909.34	242.43	<5.11	<5.67	<0.66	<3.11	<20.11	4.77
630.07	54.13	1,805.00	1,579.00	<5.11	<5.67	<0.66	<3.11	<20.11	9.69
671.50	24.73	3,318.00	41.14	<5.11	21.12	<0.66	<3.11	<20.11	5.91
1,658.00	24.73	924.12	69.53	<5.11	<5.67	<0.66	<3.11	<20.11	5.33
587.95	24.73	923.34	38.93	<5.11	<5.67	<0.66	<3.11	<20.11	8.38
501.55	14.06	452.46	47.74	<5.11	<5.67	<0.66	<3.11	<20.11	<4.51
712.28	14.06	1,336.00	41.14	<5.11	21.12	<0.66	<3.11	<20.11	5.33
830.95	14.06	821.95	64.14	<5.11	<5.67	<0.66	<3.11	<20.11	21.25
869.37	24.73	1,749.00	192.55	<5.11	<5.67	<0.66	<3.11	<20.11	<4.51
630.07	36.18	>3,860.00	172.07	<5.11	21.12	<0.66	<3.11	<20.11	10.36

1,441.00	14.06	1,029.00	35.64	<5.11	<5.67	<0.66	<3.11	<20.11	<4.51
1,878.00	36.18	1,356.00	71.68	<5.11	<5.67	<0.66	<3.11	<20.11	12.42
1,425.00	14.06	877.54	17.45	<5.11	<5.67	<0.66	<3.11	<20.11	<4.51
342.86	24.73	737.61	58.70	<5.11	<5.67	<0.66	<3.11	<20.11	14.55
630.07	24.73	446.40	65.22	<5.11	<5.67	<0.66	<3.11	<20.11	<4.51
319.38	24.73	2,070.00	279.11	<5.11	<5.67	<0.66	<3.11	<20.11	4.77
1,361.00	48.08	1,693.00	209.86	<5.11	<5.67	<0.66	<3.11	<20.11	<4.51
366.13	24.73	3,526.00	47.74	<5.11	<5.67	<0.66	<3.11	<20.11	<4.51
869.37	24.73	2,932.00	113.14	<5.11	<5.67	<0.66	<3.11	<20.11	7.12
366.13	24.73	720.81	490.67	<5.11	21.12	<0.66	<3.11	<20.11	<4.51
630.07	14.06	1,596.00	38.93	<5.11	<5.67	<0.66	<3.11	<20.11	<4.51
1,018.00	24.73	1,662.00	63.05	<5.11	<5.67	<0.66	<3.11	<20.11	<4.51
1,211.00	36.18	1,671.00	1,385.00	<5.11	<5.67	<0.66	<3.11	<20.11	<4.51
791.97	24.73	1,030.00	82.32	<5.11	<5.67	<0.66	<3.11	<20.11	<4.51
1,228.00	24.73	>3,860.00	30.17	<5.11	<5.67	<0.66	<3.11	<20.11	6.51
587.95	24.73	1,939.00	54.33	<5.11	<5.67	<0.66	<3.11	<20.11	<4.51
1,878.00	14.06	1,555.00	54.33	<5.11	<5.67	<0.66	<3.11	<20.11	<4.51
<157.64	<9.33	2,077.00	38.93	<5.11	<5.67	<0.66	<3.11	<20.11	<4.51
1,160.00	157.67	521.31	323.16	<5.11	<5.67	22.23	31.97	<20.11	<4.51

Supplemental Table S3. Antibodies.

Antibody	Supplier	Catalog # or clone	Application
CD68	Agilent	clone KP1	IHC
S100A6	Thermo Fisher Scientific	H00006277-M16	IHC
NFκB	Cell Signaling	8242	WB
pNFκB	Cell Signaling	3039	WB
STAT3	Cell Signaling	4904	WB
pSTAT3	Cell Signaling	9131	WB
IκBα	Cell Signaling	9242	WB
IKKα	Cell Signaling	2682	WB
pIKKα/β	Cell Signaling	2694	WB
SIRT3	Cell Signaling	2627	WB
JNK	Cell Signaling	9252	WB
pJNK	Cell Signaling	9251	WB
MnSOD	Enzo Life Sciences	ADI-SOD-110	WB
Ac-MnSOD	Gift from Dr. Gius	clone K68	WB
Ac-p53	Cell Signaling	2525	WB
CD3 PE	BD Biosciences	556612	FC
CD20 BV510	Biolegend	302340	FC
CD16 Pacific Blue	Biolegend	302032	FC
CD11c APC	Biolegend	301614	FC
CD14 Alexa Fluor 700	Biolegend	301822	FC
CD123 PerCP/Cy5.5	Biolegend	306016	FC
CD8a PE/Cy7	Biolegend	301012	FC
HLA-DR APC/Cy7	Biolegend	307618	FC
CD8b ECD	Beckman Coulter	6607123	FC
CD4 PerCP/Cy5.5	Tonbo Biosciences	65-0048	FC
CD27 APC	Tonbo Biosciences	20-0279	FC
CCR7 PE/Cy7	Biolegend	353226	FC
CD28 PE	Biolegend	302908	FC
CD95 Pacific Blue	Biolegend	305619	FC
IgD-biotin*	SouthernBiotech	2030-08	FC

*Paired with Alexa Fluor 700 streptavidin (Thermo, catalog #S21383).
FC, flow cytometry; IHC, immunohistochemistry; WB, Western blot.

Supplemental Table S4. qPCR primer sequences.

Gene	Forward Primer	Reverse Primer
ACTA2	GGCAAGTGATCACCATCGGA	GTGGTTTCATGGATGCCTGC
AKAP12	AAACGGCCAGGAGGAAGAAG	GTGACGTCTTGAACAACCGC
B2M	CGAGATGTCTCGCTCAGTGG	CATTCTCTGGTGGATGGCGT
CCR2	TTGACGTGAAGCAAATCGGG	CCAGCATGTTGCCCACAAAA
CD11b (ITGAM)	ACGCGCAGGCAGACGAAGAC	TCAGGCGCAGCACAATGGGG
COL1A1	AAGGACAAGAGGCACGTCTG	CAGGAAGGTCAGCTGGATGG
COL3A1	GGTCCAAAGGGTGACAAGGG	GGCCAGGAGGACCAATAGGA
EZH2	AGGATGTGGACACTCCTCCA	CCGTGGATGATCACAGGGTT
FAP	AGTTTCAGCGACTACGCCAA	TCTTGATCAGTGCGTCCGTC
HMBS	GCCCAGCTGCAGAGAAAGTT	GATGGCACTGAACTCCTGCT
ICAM1	AACCCTCCTCACCGTGTACT	GGCAGCGTAGGGTAAGGTTC
IL10	GACCTCCGAGATGCCTTCAG	CTCAGACAAGGCTTGGCAAC
IL12B	GCAGCTGGTCATCTCTTGGT	CACCATTTCTCCAGGGGCAT
IL1B	GCTCTCCACCTCCAGGGACAGG	TGAGGCCCAAGGCCACAGGT
IL6	TCCTCGACGGCATCTCAGCC	TGCCAGTGCCTCTTTGCTGCT
LGALS3	ACAATTCTGGGCACGGTGAA	CGTGGGTTAAAGTGGAAGGC
MCP1 (CCL2)	GCACTTCTGTGCCTGCTGCTCA	GCAGCAAGTGACGGGGGCAT
NFKBIA	CGGAGTTCACGGAGGATGAG	GTTCTTTCAGCCCCTTTGCG
PDGFRB	CTTCCATGAGGACGCTGAGG	CTGGGTGGCTCTCACTTAGC
PGDFA	CCTGCCCATTCGAAGGAAGA	CAGATCAGGAAGTTGGCGGA
RPS15	TTTCTGAGCATCCGGCAAGA	CATCAGCTGCTCGTAGGACAT
SOCS3	GGCTCAGCCCCAAGGACGGA	TGGAGCCAGCGTGGATCTGC
TGFB1	GAGCCCTGGACACCAACTAC	GAGGTCCTTGCGGAAGTCAA
TLR4	GCTTCCTCCGTTTTCCAGAACTGC	TGGAGAGGTGGCTTAGGCTCTGA
TNF	TCGAACCCCAAGTGACAAGCCT	GCCATTGGCCAGGAGGGCAT
TNFSF12	GTTCATCCACGACCTGGACA	GGGCTGGAGCTGTTGATTCT
TREM2	ATCTACAACCCCACGATGCG	CAGAGATCTCCAGCATCCCG
VCAM1	CTGTGCCCACAGAAAGGGAG	CATGGTCACAGAGCCACCTT
VEGFA	CCCACTGAGGAGTCCAACAT	CTCCTATGTGCTGGCCTTGG
WWTR1	GGCTGGGAGATGACCTTCAC	GGCTGATTCATCGCCTTCCT
